# Polyzwitterionic Organohydrogel and Soft Composite with Tunable Sol–Gel Properties Enabling On‐Demand Functionalization with Colloids

**DOI:** 10.1002/advs.202518531

**Published:** 2025-12-14

**Authors:** Ziyue Miao, Xiaodan Hong, Olli Ikkala, Zhong‐Peng Lv, Bo Peng

**Affiliations:** ^1^ College of Smart Materials and Future Energy State Key Laboratory of Coatings for Advanced Equipment and Advanced Coating Research Center of Ministry of Education of China Fudan University Shanghai 200433 China; ^2^ Department of Applied Physics Aalto University Aalto Espoo FI‐00076 Finland; ^3^ Shenzhen Institutes of Advanced Technology Chinese Academy of Sciences Shenzhen 518055 China

**Keywords:** electromagnetic interference shielding, mechano‐responsive function, organohydrogels, sol–gel transition, transducer, viscoelasticity, Zwitterionic polymer

## Abstract

While zwitterionic hydrogels and aq. polymers have already been used in, e.g., bio‐related, environmental, and ionic transport‐related applications, it is foreseen that their characteristic ability of zwitterions to bind functional particles combined with sol–gel transitions can allow emerging potential for responsive soft composites. Here, it is first shown that polyzwitterionic poly[2‐(methacryloyloxy)ethyl]dimethyl‐(3‐sulfopropyl)ammonium hydroxide (PDMAPS), allows organohydrogelation upon adding dimethyl sulfoxide to its aqueous solution, inducing phase‐separations to form physical cross‐links. Density functional theory (DFT) analysis reveals solvent–polymer interactions that drive the organohydragelation. The organohydrogels exhibit ultrahigh stretchability (>2800%), quick self‐adhesion, remoldability, and tunable viscoelasticity. By modulating solvent composition and integrating functional fillers, distinct sol‐ and gel‐like states are achieved on‐demand. In the sol‐like state, titanium carbide nanosheets (MXenes)‐incorporated PDMAPS soft composite enable mechano‐tunable electromagnetic interference shielding via nanosheet reorientation under strain. In the gel‐like regime, incorporation of magneticneodymium iron boron magnet (NdFeB) microparticles yields mechano–magneto–electric transducers for strain detection, dynamic haptic functionality, as demonstrated by Morse code encoding, and high durability in repeated compressive cycles. This work introduces a versatile organohydrogel platform with tunable viscoelastic properties, suitable for on‐demand functionalized soft composites, suggesting new design principles for transducing, sensing, and soft robotics.

## Graphical abstract

Poly[2‐(methacryloyloxy)ethyl]dimethyl‐(3‐sulfopropyl)ammonium hydroxide‐based organohydrogels are formed via nonsolvent‐induced gelation, featuring tunable viscoelasticity. Functional fillers (MXene nanosheets, magnetic NdFeB microparticles) enable electrical, magnetic, and mechanical responsiveness for applications in mechano‐tunable electromagnetic interference shielding and mechano–magneto–electric transducers for strain sensing, offering a versatile platform for adaptive, multifunctional soft composites.

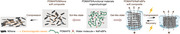

## 1 Introduction

Polymers in solvent that exist in the sol and gel states and their soft composites upon functionalization with nano‐ or microscale particles are attractive for diverse applications.^[^ ^1^, ^2^, ^3^ ^]^ In the sol state, they behave as viscous fluids, enabling plastic deformations and facilitating reversible shaping through casting, (re‐)molding, or printing. Upon transition to the gel state, a percolated 3D network forms, allowing for elastic deformations and shape retention under moderate stress.^[^ ^4^, ^5^, ^6^ ^]^ Such materials are increasingly appreciated for their potential in multifunctional systems that allow on‐demand switching between reconfigurable and structurally stable phases.^[^ ^7^, ^8^ ^]^

Among polymers allowing gels, polyzwitterionic polymers have attracted significant interest due to their combination of high hydration capacity, antifouling properties, and bio‐compatibility.^[^ ^9^, ^10^, ^11^, ^12^ ^]^ In particular, poly[2‐(methacryloyloxy)ethyl]dimethyl‐(3‐sulfopropyl) ammonium hydroxide (PDMAPS) stands out feasible due to its biocompatibility and it has been studied, e.g., for wound healing,^[^ ^13^, ^14^ ^]^ ionic conduction,^[^ ^15^, ^16^ ^]^ solar desalination^[^ ^17^, ^18^ ^]^ and sensing.^[^ ^19^, ^20^, ^21^, ^22^, ^23^, ^24^ ^]^ Therein, the existing research have primarily focused on the gel state, while the tunability of sol–gel transitions in such systems remains largely underexplored. Most zwitterionic hydrogels exhibit limited^[^ ^24^, ^25^, ^26^ ^]^ or thermoresponsive sol–gel behavior only at low polymer concentrations.^[^ ^27^, ^28^ ^]^ In contrast, our PDMAPS‐based organohydrogels exhibit a solvent‐induced sol–gel transition hat operates at high polymer concentrations, enabling tunable viscoelasticity and multifunctionality not attainable in previously reported zwitterionic systems.

Herein, PDMAPS‐based organohydrogels are first synthesized via aqueous polymerization, followed by non‐solvent‐induced phase separation (NIPS) using dimethyl sulfoxide (DMSO). They exhibit high stretchability, self‐adhesion, re‐moldability, and most notably, tunable sol–gel behavior. By modulating the viscoelastic properties through adjustments of additional organic solvent type and content, and polymer concentration, versatile soft composites are allowed based on incorporating further functional constituents, such as titanium carbide nanosheets (MXenes, Ti_3_C_2_T_x_)^[^ ^29^, ^30^ ^]^ and neodymium iron boron magnet particles (NdFeBPs).^[^ ^31^, ^32^ ^]^ The engineered sol–gel transition not only governs the mechanical tunability and responsiveness of the composites but also imparts structural programmability and long‐term stability, making them useful candidates for applications in electromagnetic interference (EMI) shielding and self‐powered mechano–magneto–electric transduction for sensing.

## 2 Results and Discussion

### 2.1 PDMAPS Soft Composites: Design and Characterization

**Figure 1:**
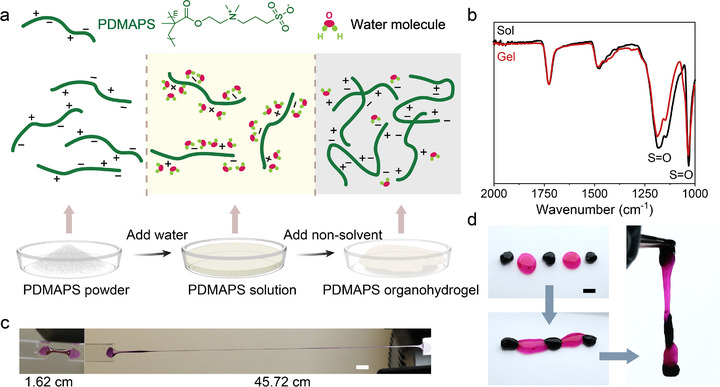
Characterization of PDMAPS‐based organohydrogels. a) Schematic illustration of the preparation of PDMAPS organohydrogels. b) FTIR spectra (in the regions of 2000–1000 cm^−1^) of the freeze‐dried PDMAPS and PDMAPS organohydrogels. c) Photographs of the PDMAPS organohydrogels before and after stretching to 2820%. (Scale bar: 2 cm) d) Demonstration of self‐adhesive ability (dyed by rose bengal (pink) and methylene blue (blue)). (Scale bar: 1 cm).

PDMAPS is synthesized via free radical polymerization of the DMAPS monomer, followed by mechanical grinding into powders to facilitate improved dissolution in water (**Figure** **1**a). The chemical structure and composition of the resulting polymer were verified using proton nuclear magnetic resonance spectroscopy (^1^H NMR; Figure S1, Supporting Information), while scanning electron microscopy (SEM; Figure S2, Supporting Information) suggests the assembled morphology.

To prepare the organohydrogels, PDMAPS powder is first dissolved in water, and the resulting concentrated aqueous PDMAPS solution is equilibrated for three days prior to the addition of DMSO to ensure complete polymer hydration and chain relaxation.^[^ ^33^, ^34^ ^]^ This equilibration process yields a homogeneous precursor state essential for forming reproducible organohydrogels with consistent properties. Subsequently, DMSO is introduced as a non‐solvent to induce gelation. Gelation is driven by changes in solvent–polymer interactions upon DMSO addition. DMSO competes with water for interactions with the zwitterionic polymer, effectively dehydrating the polymer chains. This promotes polymer–polymer associations via zwitterionic interactions, leading to the formation of a physical gel network (Figure 1a; Movie S1, Supporting Information). Fourier‐transform infrared (FTIR) spectroscopy of the freeze‐dried samples is employed to elucidate the structural changes induced by DMSO (Figure 1b). Compared to pristine PDMAPS, the intensities of both asymmetric (≈1152 cm^−1^) and symmetric (≈1040 cm^−1^) S═O stretching vibrations of the organogels show noticeable decrease in the gel state, while the asymmetric stretch peak simultaneously exhibits a blueshifting trend. These spectral modifications indicate a dehydration process of sulfonate groups and subsequent intensified ionic interactions between the sulfonate and ammonium moieties.^[^ ^35^, ^36^ ^]^ SEM analysis further corroborates this interpretation, suggesting a reduction in pore size within the network (Figures S2 and S3, Supporting Information), indicative of increased structural densification.

The resulting PDMAPS organohydrogels demonstrate remarkable mechanical extensibility with a maximum elongation of 2820% (Figures 1c; S4, Supporting Information), and quick self‐adhesion within 5 s upon re‐joining separates pieces (Figures 1d; S5 and Movie S2, Supporting Information). These properties are attributed to the absence of strong covalent cross‐links, allowing for flexible chain mobility and dynamic interactions. Critically, these gels exhibit excellent long‐term stability, retaining over 95.1 wt.% of their solvent content and maintaining their mechanical properties (*G′* and *G″*) over one week without phase separation (Figure S6, Supporting Information). Moreover, the physically cross‐linked nature of the composites affords re‐moldability: the composite can be dissolved in water, lyophilized to regenerate PDMAPS powder, and reconstituted by reintroducing water and DMSO in the original ratios (Figure S7, Supporting Information).

### 2.2 Tunable Sol–Gel Properties of the Organohydrogels

**Figure 2:**
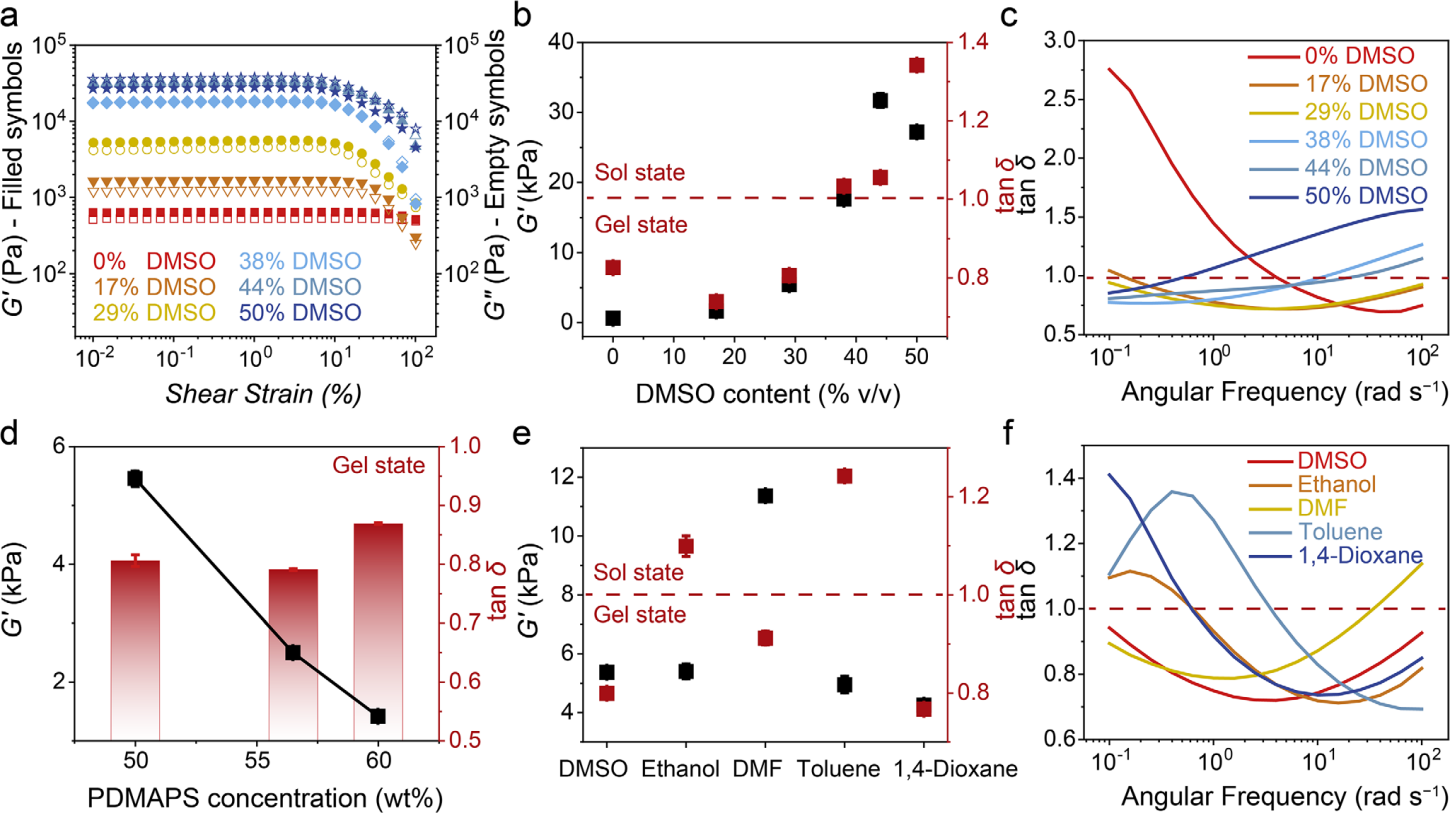
Rheological characterization of PDMAPS organohydrogels. a) Strain amplitude sweep curves for samples containing varying DMSO concentrations (%, v/v). b) Dependence of *G′* and tan *δ* on DMSO content (%, v/v), determined within the linear viscoelastic region from (a). c) Frequency‐dependent behavior of tan *δ* for samples with different DMSO contents (%, v/v). d) Variation in *G′* and tan *δ* as a function of PDMAPS concentration (wt.%), measured within the linear viscoelastic region from Figure S9 (Supporting Information). e) Variation of *G′* and tan *δ* of samples with different non‐solvents measured from linear viscoelastic region in amplitude sweep measurements from Figure S10 (Supporting Information). f) Frequency‐dependent behavior of tan *δ* for samples with different non‐solvents. The data in (b,d,e) are expressed as mean ± standard error (SD) (*n* = 3 independent samples).

The rheological properties of PDMAPS organohydrogels were investigated to elucidate how the non‐solvent content, polymer concentration, and solvent identity modulate the mechanical properties. Amplitude sweep measurements (**Figure** **2**a) reveal that increasing DMSO concentrations of DMSO (0%, 17%, 29%, 38%, 44%, and 50% v/v relative to the total solution volume) first enhance both the storage (*G′*) and loss (*G′′*) moduli, suggesting increased mechanical strength. However, a decline in *G′* is observed at 50% DMSO, indicating that excessive non‐solvent disrupts the network structure.

The loss factor (tan *δ* = *G′′*/*G′*), plotted in Figure 2b, increases with DMSO content, signifying a gradual transition from a gel‐like state (tan *δ* <1) to a more liquid‐like behavior (tan *δ* >1). This trend suggests that higher DMSO concentration weakens the electrostatic interactions between the zwitterionic polymer chains, thereby compromising the integrity of the physically cross‐linked network. Frequency sweep data (Figures 2c; S8, Supporting Information) further confirm that at 29% DMSO, the composition maintains tan *δ* <1 over the entire frequency range (10^−1^–10^2^ rad s^−1^), indicating stable gel behavior. Although higher DMSO levels may enhance rigidity, they also destabilize the network under dynamic deformation. Thereby, DMSO was chosen at 29 % (v/v) for further studies to ensure a robust and stable gel network.

The effect of polymer concentration is investigated by varying PDMAPS concentration (50, 56.5, and 60 wt.%) at a fixed DMSO content of 29% (v/v). As shown in Figures 2d and S9 (Supporting Information), *G′* increases with polymer concentration, indicating enhanced cross‐link density and network stiffness. Across all concentrations, tan *δ* remains below 1, signifying dominance of elastic over viscous behavior and confirming the formation of robust physical gels.

To explore the gelation capability, different organic solvents were added, i.e., DMSO, ethanol, *N*,*N*‐dimethylformamide (DMF), toluene, and 1,4‐dioxane, using a fixed PDMAPS content (50 wt.%) and constant solvent fraction (29%). Amplitude sweep results (Figures 2e; S10, Supporting Information) reveal successful gelation (tan* δ* <1) for composites formed with DMSO, DMF, and 1,4‐dioxane. Among them, only DMSO‐based gels maintained tan *δ* <1 across the entire frequency range (Figures 2f; S11, Supporting Information). These findings indicate that only DMSO enables the formation of a structurally stable and predominantly elastic gel network, highlighting its unique ability—compared with other tested solvents—to produce tunable and mechanically resilient organohydrogels.

### 2.3 DFT Insights into Solvent‐Polymer Interactions

**Figure 3:**
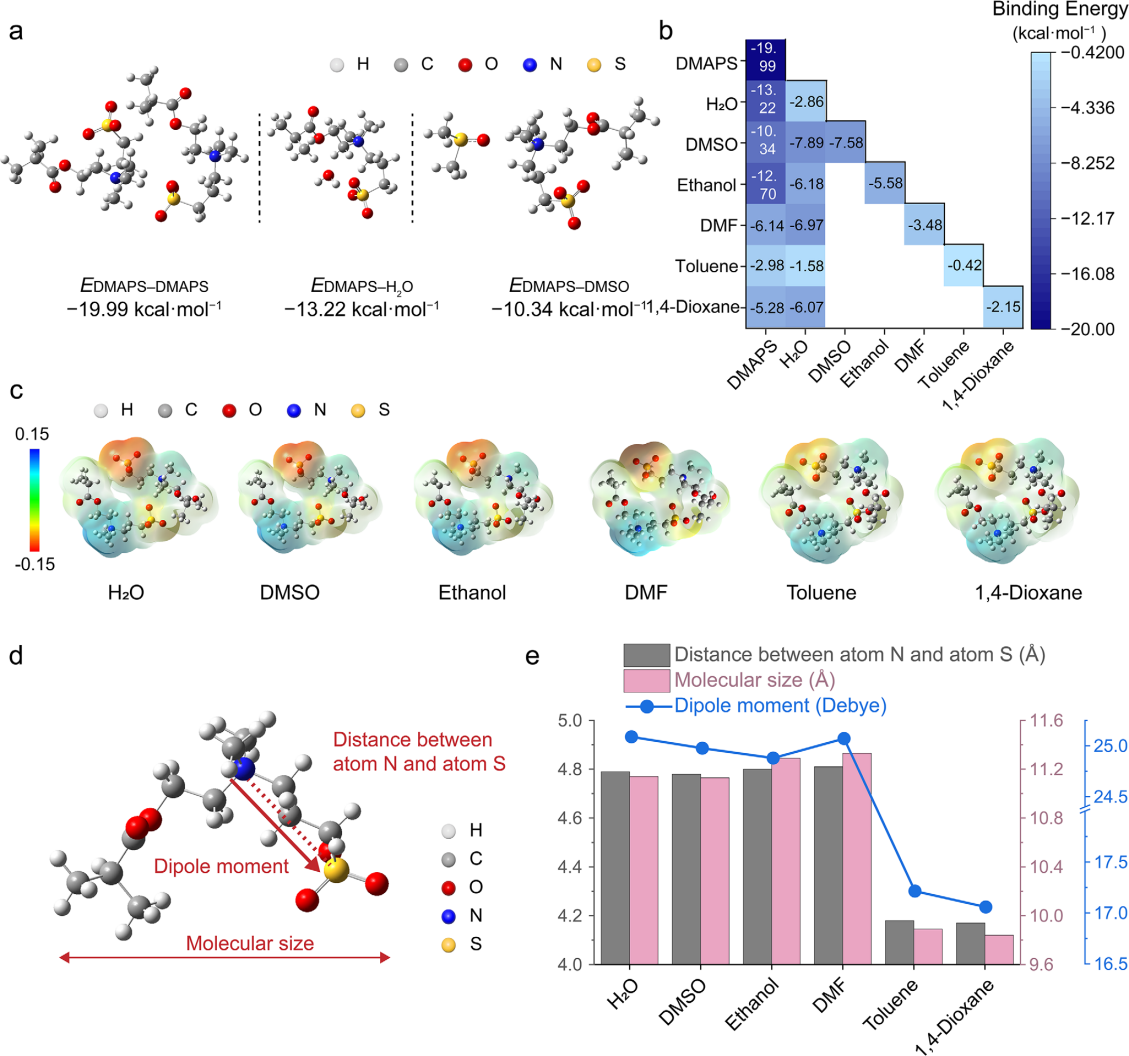
Theoretical exploration of the molecular interactions. a) DFT calculations of the DMAPS–DMAPS, DMAPS–water, and DMAPS–DMSO interactions. b) Summary of DFT binding energies between DMAPS, water and different non‐solvents. c) EPSs of the optimized DMAPS–DMAPS structure in different non‐solvent atmospheres. d) Illustration of the optimized DMAPS in solvent. Red dashed line indicates the distance between N and S atoms; red vector represents the dipole moment; red double vector denotes the molecular size. e) Distances between atoms N and S, molecular sizes, and dipole moments of optimized DMAPS structures in different solvent atmospheres.

To gain molecular‐level insight into the sol–gel behavior of PDMAPS organohydrogels, density functional theory (DFT) calculations were conducted to probe the interactions between the DMAPS monomer and different solvents, including DMSO, ethanol, DMF, toluene, and 1,4‐dioxane. As shown in **Figure** **3**a, DMAPS monomer forms strong intermolecular interactions (e.g., hydrogen‐Bonding and electrostatic interactions) with both water and itself. Upon addition of DMSO, significant interactions are established between DMSO and DMAPS, enabling DMSO to integrate into the polymer network and facilitate chain aggregation (Figure 3a).

Binding energy calculations (Figure 3b) reveal that DMSO interacts more strongly with DMAPS than with water, allowing it to displace water from the hydration shell of the polymer and thus to promote network densification through increased chain–chain interactions. This shift in DMAPS–DMAPS, DMAPS–water, and DMAPS–DMSO interactions drives controlled phase separation and the formation of dynamic physical cross‐links, thereby supporting the observed solvent‐induced sol–gel transition and tunable viscoelasticity.

In contrast, DMF and 1,4‐dioxane exhibit preferential binding with water rather than with DMAPS (Figure 3b), allowing them to extract water from the system and induce solvent plasticization. This mechanism explains the formation of stable gels in these solvents (*δ* <1 in Figure 2e). Moreover, the binding energy of DMF–water (−6.97 kcal·mol^−1^) exceeds that of 1,4‐dioxane–water (−6.07 kcal·mol^−1^), aligning with the higher *G′* observed in DMF‐based composites (Figure 2e).

Ethanol and toluene exhibit distinct interaction profiles. Ethanol shows strong affinity for DMAPS (−12.7 kcal·mol^−1^), greater than its interactions with water (−6.18 kcal·mol^−1^) or itself (−5.58 kcal·mol^−1^). Instead of displacing water, ethanol integrates into network and competes for binding sites, effectively increasing the total solvent content and enhancing molecular mobility. This behavior results in tan *δ* >1 and a more fluid‐like material (Figure 2e).

Toluene, a low‐polarity and water‐immiscible solvent, exhibits a weak binding energy to DMAPS (−2.98 kcal·mol^−1^), and thus fails to disrupt existing DMAPS−DMAPS (−19.99 kcal·mol^−1^) and DMAPS−water (−13.22 kcal·mol^−1^) interactions, leading to sparse physical cross‐links and diminished gel mechanical strength. The weak cross‐linking is further confirmed by rheological measurements (Figure 2e), showing a fluid‐like behavior (tan *δ* >1). These findings underscore the crucial role of solvent polarity and miscibility in organohydrogel formation.

Collectively, these DFT results corroborate the rheological data in Figure 2e, clarifying the distinct mechanisms by which each solvent modulates network assembly. Among all tested solvents, DMSO stands out as an effective non‐solvent, due to its strong, dual binding affinity for both DMAPS (second only to ethanol) and water (the best of all solvents in the present case), enabling it to facilitate chain aggregation while stabilizing the solvent environment, key to achieving optimal gel strength and durability.

To further probe these solvent‐specific effects, electrostatic potential surfaces (EPSs) of the optimized DMAPS–DMAPS structures were computed in the presence of each solvent (Figure 3c). While the EPS around the ammonium groups remains relatively unchanged, substantial variations are observed at the sulfonate site. A shift from darker to lighter EPS coloration corresponds to a decrease in local charge density, following the order: water >DMSO >ethanol >DMF >toluene >1,4‐dioxane. Increased sulfonate charge density enhances intermolecular electrostatic repulsion and dispersion, thereby inhibiting aggregation and promoting stable dispersion within the composite.

Molecular descriptors including the N–S atomic distance, molecular size, and dipole moment are extracted from the optimized DMAPS geometries under each solvent condition (Figure 3d, summarized in Figure 3e). Notably, the trends for anion‐cation distance and molecular size exhibit a slightly different trend compared to the sequence observed in Figure 3b,c. In both DMF and ethanol, DMAPS exhibits slightly larger anion–cation distances and molecular dimensions compared to water and DMSO. This can be attributed to the polar functional groups present in DMF and ethanol. DMF's polar amide group interacts with the sulfonate moiety of DMAPS, weakening the intramolecular electrostatic attraction. Similarly, ethanol, as a protic solvent, forms hydrogen bonds with DMAPS, partially shielding its zwitterionic charges. In contrast, significantly shorter anion–cation distances are observed in toluene and 1,4‐dioxane due to their low dielectric constants and poor polarity, which provide insufficient electrostatic stabilization.

Lastly, the molecular dipole moment (*µ*), a measure of charge separation within the molecule, is presented as red vectors in Figure 3d and quantified in Figure 3e. The dipole moment (*µ* = *q* × *r*, where *q* is the charge and *r* is the separation distance) reflects the polarity of DMAPS in each solvent environment. DMAPS displays the highest dipole moments in water and DMSO (with the exception of DMF), signifying strong charge separation and thus high molecular polarity. This trend aligns well with the interaction and EPS analyses, except for DMF, whose unique solvation behavior introduces a minor discrepancy.

Overall, DFT calculations reveal that DMSO's strong interactions with DMAPS and water facilitate controlled phase separation, chain aggregation, and dynamic cross‐linking, whereas other solvents either compete with DMAPS for water or fail to interact sufficiently with the polymer. These molecular‐level insights explain the superior gel stability and multifunctionality observed in DMSO‐based organohydrogels.

### 2.4 PDMAPS Soft Composites Based on Organohydrogels Upon Adding Functional Particles

**Figure 4:**
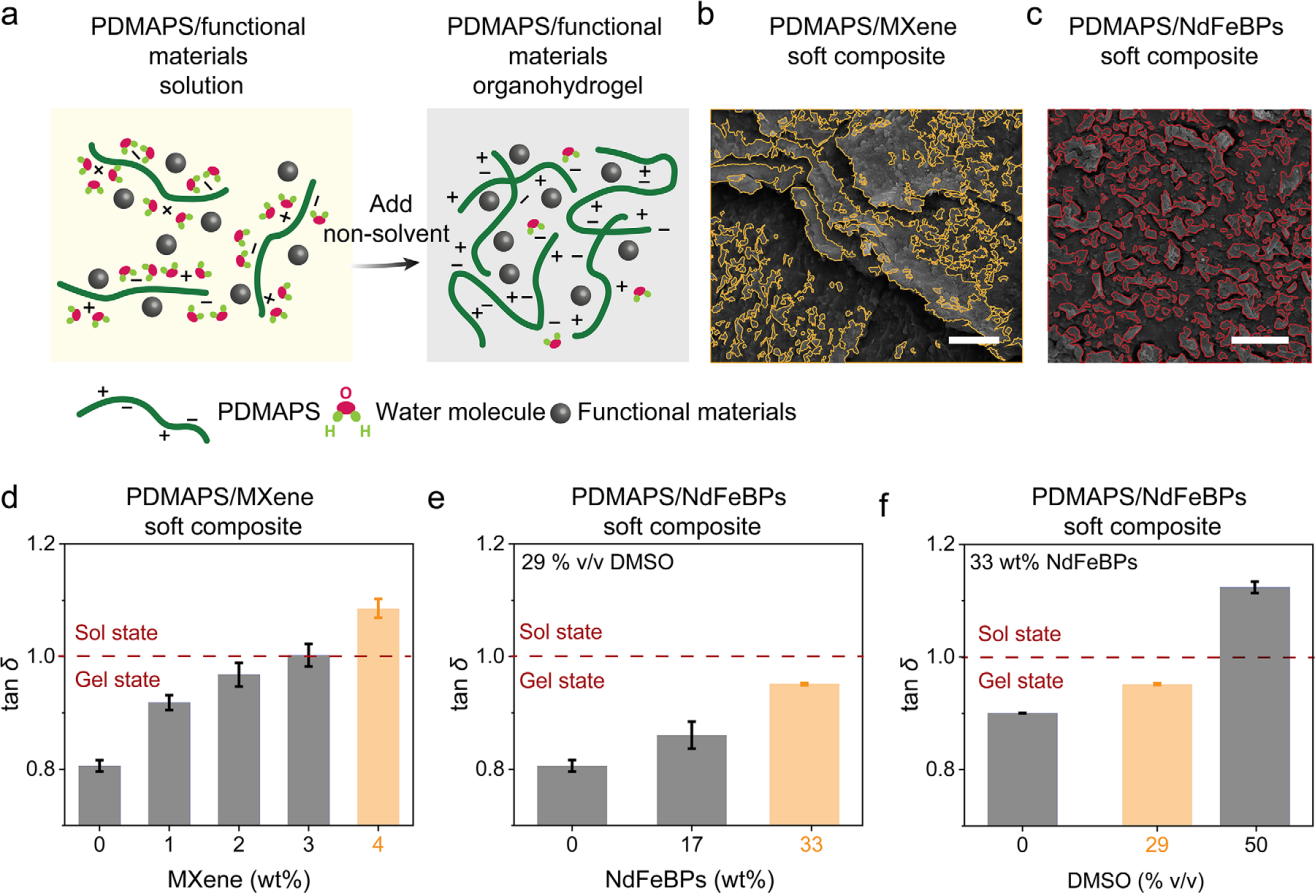
Functional soft composites based on PDMAPS organohydrogels. a) Schematic diagram of NIPS‐induced PDMAPS/functional filler soft composite preparation. b,c) SEM images of PDMAPS/MXene (with MXene circled by yellow line) and PDMAPS/NdFeBPs soft composite (with NdFeBPs circled by red line). Scale bar 4 µm. d) tan *δ* of PDMAPS/MXene composites with different MXene concentrations, measured within the linear viscoelastic region from Figure S13 (Supporting Information). e) tan *δ* of and PDMAPS/NdFeBPs soft composite containing 29 % (v/v) DMSO with different NdFeBPs contents (wt.%) measured in the linear viscoelastic region from Figure S14 (Supporting Information). f) tan *δ* of and PDMAPS/NdFeBPs soft composite containing 33 wt.% NdFeBPs with different DMSO contents (% v/v) measured in the linear viscoelastic region from Figure S15 (Supporting Information). The orange columns indicate the samples selected for further application in Sections 2.4.1 and 2.4.2. The orange‐highlighted soft composites in (e,f) represent the same formulation. The data in (d,e,f) are expressed as mean ± SD (*n* = 3 independent samples).

PDMAPS‐based organohydrogels can be functionalized by incorporating additional nanomaterials during the initial preparation phase to allow functional soft composites, see **Figure** **4**a for the schematics. To demonstrate the generality, two different types of particles were explored, electrically conducting MXenes and magnetic NdFeBPs. Both allow homogeneous mixing within the aq. PDMAPS phase followed by organohydrogelation upon adding DMSO (Figure 4b,c). Polystyrene (PS) microspheres were used as tracer particles under the same mixing and gelation conditions. The resulting SEM image (Figure S12, Supporting Information) shows uniformly distributed particles, indicating that MXene and NdFeBPs are similarly homogeneously dispersed within the PDMAPS organohydrogel.

The sol–gel behavior of the PDMAPS/MXene and PDMAPS/NdFeBPs soft composites can be precisely modulated by adjusting the MXene content (Figures 4d; S13, Supporting Information), NdFeBPs loading (Figures 4e; S14, Supporting Information), and DMSO concentration (Figures 4f; S15, Supporting Information). When exhibiting liquid‐like behavior, the PDMAPS/MXene undergoes plastic deformation upon compression, enhancing conductive alignment and EMI absorption.^[^ ^37^, ^38^ ^]^ In contrast, the PDMAPS/NdFeBPs soft composites, with solid‐like behavior, exhibits elastic deformation, which ensures stable mechanical–magnetic coupling and enables reversible current generation after deformation.^[^ ^39^, ^40^, ^41^ ^]^ These details are further discussed in Sections 2.4.1 and 2.4.2.

#### 2.4.1 EMI Shielding Soft Nanocomposites Based on Organohydrogel Hybrids with MXene Nanosheets

**Figure 5:**
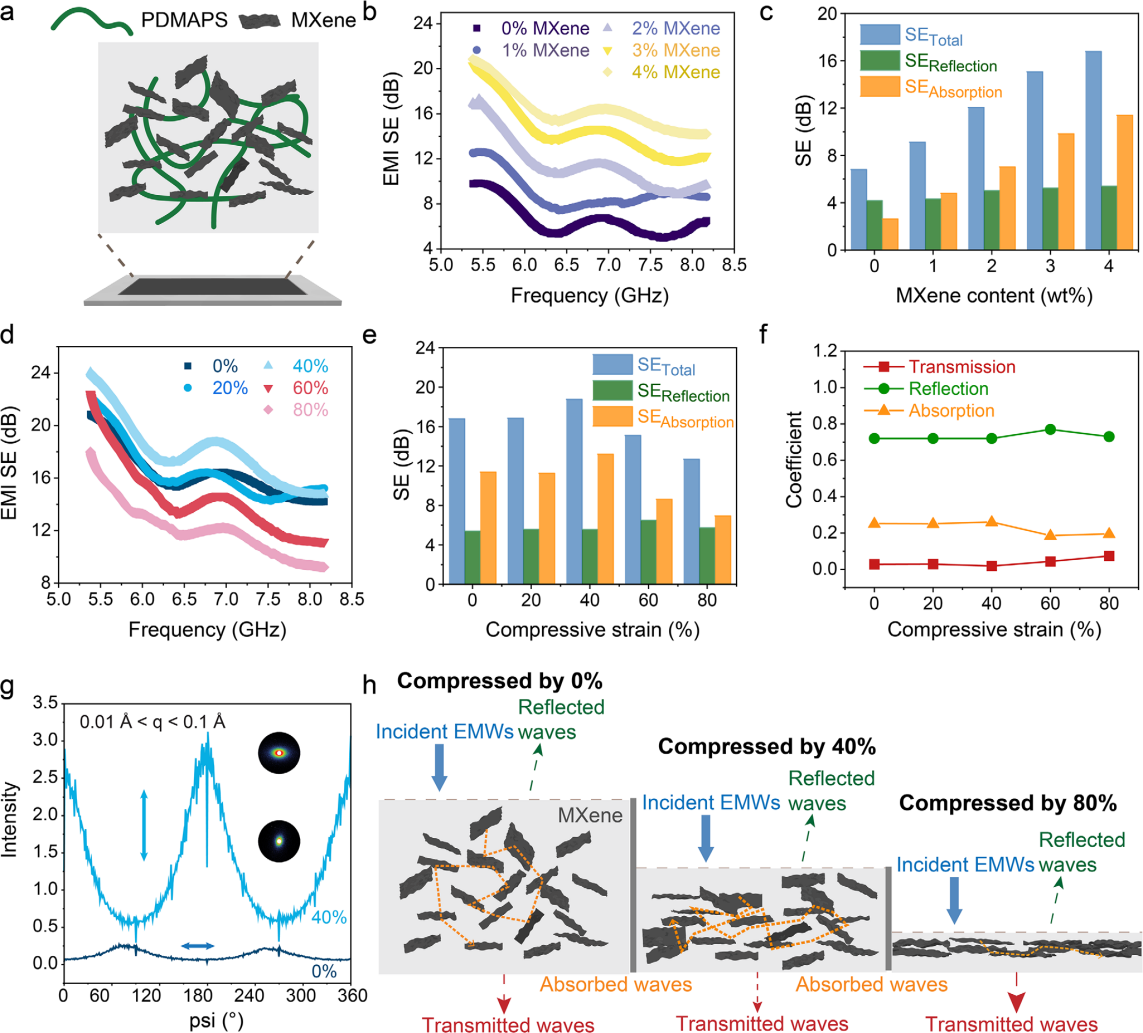
EMI shielding performance of PDMAPS/MXene soft composites. a) Schematic illustration of a PDMAPS/MXene soft composite positioned within a 1 mm‐thick mold for EMI shielding measurements. b,c) The EMI shielding efficiency (SE) (b) and SE_Total_, SE_Reflection_, SE_Absorption_ values (c) of PDMAPS/MXene composites as a function of MXene content. d–f) EMI shielding characteristics of PDMAPS/MXene (4 wt.% MXene) under different compressive strains: EMI SE (d), SE_Total_, SE_Reflection_, SE_Absorption_ values (e), and power coefficients of Transmission, Reflection, Absorption (f) of PDMAPS/MXene. g) Azimuthal profiles of 0% (blue) and 40% (cyan) samples, derived from 2D SAXS/WAXS data (upper one 40%, bottom one 0%) in the *q* range between 0.01 and 0.1 Å^−1^. The double arrows indicate the MXene flake alignment directions in the samples. The double arrows indicate the MXene flake alignment directions in the samples. h) The proposed EMI shielding mechanism of PDMAPS/MXene under varying compressive strains.

PDMAPS/MXene soft nanocomposites were synthesized via direct mixing of delaminated MXene nanosheets dispersion into PDMAPS aqueous solutions prior to the non‐solvent‐induced organohydrogelation. MXenes involve abundant surface functional groups, relatively large lateral dimensions, mechanical flexibility, metallic conductivity, and they can be used for modulating electromagnetic waves in a wide band range.^[^ ^37^, ^38^ ^]^ MXene nanosheets are uniformly dispersed throughout the polymer matrix (**Figure** **5**a), yielding a multifunctional and flexible composite.

The EMI shielding effectiveness (SE) of 1 mm‐thick PDMAPS/MXene soft composites was evaluated over the 5.38–8.17 GHz frequency range (Figure 5b). The total SE (SE_Total_) increases with the MXene loading, primarily driven by an enhancement in absorption (SE_Absorption_), which rises from 2.63 to 11.39 dB as MXene content increases up to 4 wt.% (Figure 5c). Meanwhile, the reflection (SE_Reflection_) increases modestly by 1.22 dB (Figure 5c). The shielding mechanism (Figure S16, Supporting Information) reveals that the contribution from reflection increases from 57% to 72% with MXene content up to 4 wt.%, which is attributed to enhanced conductivity of PDMAPS/MXene (Figure S17, Supporting Information).

MXene incorporation can modulate the sol–gel transition behavior of the composite. As shown in Figure 4d, increasing MXene content shifts the composite from a gel‐like to a more sol‐like state due to disruption of electrostatic crosslinks within the PDMAPS network. A gel‐state PDMAPS/MXene composite (2 wt.% MXene) functions as a compressive strain sensor, exhibiting reversible resistance recovery up to 200 % strain (Figure S18, Supporting Information). In contrast, a sol‐state PDMAPS/MXene composite (4 wt.% MXene) exhibits increased storage modulus due to the intrinsic stiffness of the nanosheets, allowing plastic deformation under compression. This deformation induces reorientation and densification of MXene sheets within the polymer matrix, actively tuning the EMI shielding performance (Figure 5d–f).

Specifically, the EMI SE_Total_ of a 1 mm‐thick PDMAPS/MXene composite increases from 16.79 to 18.76 dB as compressive strain is applied up to 40% (Figure 5d), due to densification and alignment of the MXene network, which lead to an increased SE_Absorption_ from 11.39 to 13.20 dB (Figure 5e). Beyond 40%, at 80% strain, SE_Total_ drops to 12.68 dB due to collapse of the porous network and reduction in internal reflections, where SE_Absorption_ drops to 6.94 dB at 80% strain. The SE_Reflection_ remains relatively stable (5.5–6.5 dB) mainly due to the deformation does not affect much on the intrinsic conductivity. Power coefficients analysis (Figure 5f) corroborates this tunable EMI modulation behavior.

Small‐angle X‐ray scattering (SAXS) / wide‐angle X‐ray scattering (WAXS) 2D patterns (Figure S19b,c, Supporting Information) and the corresponding azimuthal intensity profiles for MXene, PDMAPS, and the PDMAPS/MXene composite (Figures 5g; S19d,e, Supporting Information) reveal distinct structural anisotropy in the compressed samples. The azimuthal profiles of the MXene lamellae (Figure S19d, Supporting Information) and the PDMAPS/MXene composite (Figure 5g) shows enhanced scattering intensity along the compression axis. The corresponding *d*‐spacing decreased from 2.4 to 2.1 nm (Figure S19f, Supporting Information), indicating structural densification. In contrast, the PDMAPS matrix itself exhibited no distinct alignment (Figure S19e, Supporting Information), confirming that the densification and directional alignment are phenomena specific to the MXene lamellae under compression. Furthermore, in the linear Porod region which corresponding to the interface of MXene and polymer matrix, the slope varied from −3 in the 0% sample to −3.3 in the 40% sample (Figure S19f, Supporting Information). This change indicates improved surface smoothness at the MXene‐polymer interface, suggesting more compact and intimate interactions after compression.^[^ ^42^ ^]^

The schematic in Figure 5h illustrates the underlying mechanism: in the uncompressed state, the porous and randomly oriented MXene structure supports multiple internal reflections. Upon application of 40% compressive strain, alignment and densification enhance wave trapping and absorption. However, excessive strain reduces thickness and structural heterogeneity, shortened propagation pathways and diminishing shielding performance.

Long‐term EMI shielding stability under static mechanical load was also evaluated. The 4 wt.% MXene composites retained >95.77 wt.% of its original mass (Figure S20a, Supporting Information) and maintained SE_Total_ with a minimal decrease, just 0.16% after one week and 12.47% after one month under constant 40% compression (Figure S20b, Supporting Information). These results underscore the remarkable mechanical and electromagnetic durability of the composite. Compared with state‐of‐the‐art MXene soft composites (Table S1, Supporting Information)^[^ ^43^, ^44^, ^45^, ^46^, ^47^, ^48^ ^]^ typically focused on static EMI shielding or strain sensing with limited durability assessment—our PDMAPS/MXene composites exhibit enhanced long‐term stability and multifunctionality, fulfilling the need for robust, multifunctional materials in soft electronics.

#### 2.4.2 Magnetic Soft Composites Based on Organohydrogel Hybrids with NdFeB Microparticles for Mechano–Magneto–Electric Transduction

PDMAPS/NdFeBPs soft composites are fabricated by dispersing magnetic NdFeBPs microparticles into the PDMAPS aqueous solution prior to the DMSO‐induced organohydrogelation. Magnetic properties of the resulting composites are evaluated as a function of NdFeBPs content and compared to pristine NdFeBPs particles (Figure S21, Supporting Information). Increasing NdFeBPs loading enhances the magnetic moment and remanence, while reducing coercivity, facilitating easier magnetization and stable magnetic retention.^[^ ^39^, ^40^, ^41^ ^]^ For example, the composite with 33 wt.% NdFeBPs shows a coercivity of 0.066 T and a remanence of 26.17 emu g^−1^, feasible for reversible magnetic actuation.

**Figure 6:**
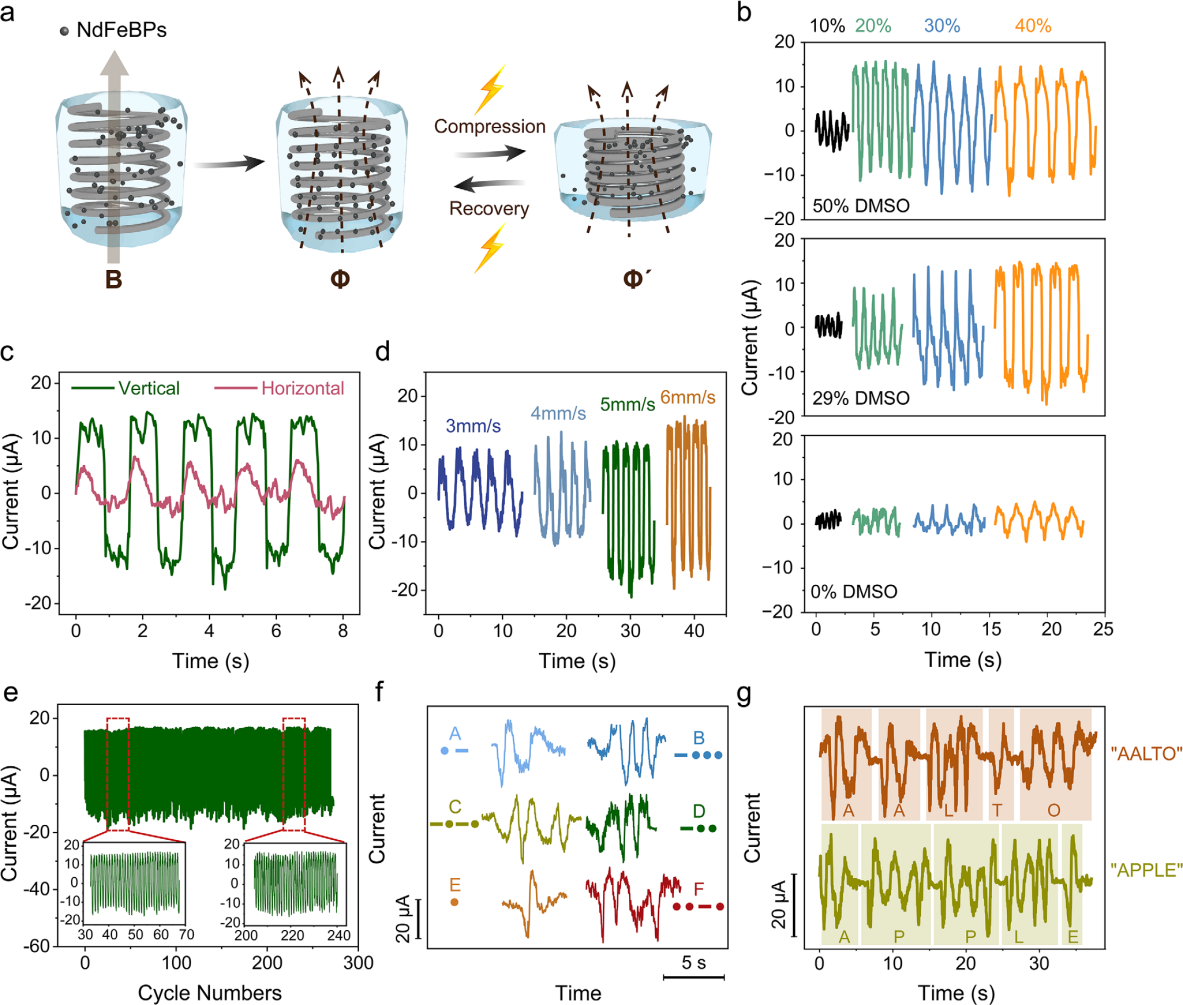
Characterization and application of PDMAPS/NdFeBPs composites. a) Schematic of the mechano–magneto–electric transduction mechanism driven by cyclic compression and recovery. b) Output current profiles of the magnetoelectric composites with different DMSO contents under various compressive strains at a constant speed of 5 mm s^−1^. c) Comparison of output currents generated under vertical and horizontal compression modes, using 40% compressive strain at 5 mm s^−1^. d) Output current plots of the magnetoelectric composite with 40% compressive strain investigated by diverse compressing velocities. e) Durability assessment of the magnetoelectric composite over 270 periodic compression–release cycles at 40% strain and 5 mm s^−1^. f) Morse code signal encoding using distinct compression speeds (6 mm s^−1^ for "dot" and 3 mm s^−1^ for "dash"). g) Real‐time output waveforms for encoded words "AALTO" and "APPLE" via magnetoelectric signal generation. All tests in (b,d–g) were conducted under vertical compression conditions.

A mechano–magneto–electric transducer is engineered by leveraging these magnetic properties.^[^ ^49^, ^50^ ^]^ As no external power source is used, we denote the system self‐powered. As illustrated in **Figure** **6**a, the device comprises a hollow cylindrical poly(hydroxyethyl methacrylate) hydrogel shell, centrally supported by a stainless‐steel spring, with the cavity filled with magnetized PDMAPS/NdFeBPs soft composites. The device is first magnetized using a variable‐gap magnetic field system (for details see Experimental Section) and subsequently compressed to generate current signals through magneto‐electric induction. The composite is designed to exhibit elastic gel‐like behavior, which facilitates rapid mechanical recovery and cyclic resilience, an essential attribute for effective electromechanical signal transduction.^[^ ^51^, ^52^ ^]^

As illustrated in Figure 4e,f, both the NdFeB loading and the DMSO concentration effectively modulate the sol–gel behavior of the PDMAPS/NdFeBPs composite. Higher NdFeB contents and lower DMSO levels enhance gel‐like characteristics and promote elastic recovery under mechanical loading. DMSO content also modulates the sensitivity of mechano–magneto–electric response (Figure 6b): while higher DMSO contents yield larger output currents under small strains (20%), they also introduce hysteresis at higher strain levels, which can hinder signal consistency. In contrast, a 29% (v/v) DMSO composition optimally balances sensitivity and response fidelity and is selected for device integration.

Under compression, the magnetized PDMAPS/NdFeBPs gel composites exhibit robust mechano–magneto–electric conversion. Current output increases with longer magnetization times, whereas unmagnetized samples show negligible signal, underscoring the essential role of remanent magnetization (Figure S22, Supporting Information). The composites exhibit directional dependence in electromechanical performance (Figure 6c), in which vertical compression produces over twice the current of horizontal loading, indicating strong anisotropy in response. A stronger current is generated when magnetic flux change is maximized within the conductive wire. Furthermore, the device accurately distinguishes between different compression velocities (Figure 6d), useful for haptics, with output current profiles showing robust reversibility and signal reproducibility across cycles. Cycling testing (Figure 6e) demonstrates robust durability, with no signal decay after 270 cycles at 40% strain and 5 mm s^−1^. Figure S23 (Supporting Information) shows that the PDMAPS/NdFeBPs composite exhibited minimal mass loss over 7 days (<3.56 wt.%) and preserved stable mechano–magneto–electric transduction under continuous deformation, further supporting long‐term structural and functional stability.

Considering its remarkable mechano–magneto–electric responses related to time encoded haptic demonstration, the composite was employed as a self‐powered Morse code transducer.^[^ ^53^, ^54^ ^]^ As shown in Figure 6f, compression velocity was modulated to encode “dot” (rapid 6 mm s^−1^) and “dash” (slow 3 mm s^−1^) compressions. The resulting current signals exhibit clear differentiation in amplitude and duration, enabling effective Morse code transmission. A full reference chart of representations from “A” to “Z” is provided in Figure S24 (Supporting Information). Figure 6g exemplifies this encoding for the words “AALTO” and “APPLE”, with output waveforms matching expected Morse sequences. These findings highlight the feasibility of PDMAPS/NdFeBPs gel composite for tactile communications and information transmission applications.

## 3 Conclusion

In this work, we first present a versatile class of PDMAPS‐based organohydrogels that combine ultra‐stretchability, intrinsic self‐adhesion, and remoldability, fabricated efficiently via a NIPS strategy. A central feature of this material platform is its tunable sol–gel behavior, which serves to modulate both mechanical properties and functionalization. By adjusting the sol–gel state and incorporating functional fillers, such as MXene and NdFeBPs, diverse and programmable performance profiles can be achieved. Specifically, PDMAPS/MXene composites exhibit sol‐like viscoelasticity, enabling tunability of EMI shielding under compressive strain. The strain‐induced alignment and densification of MXene nanosheets significantly enhance electromagnetic wave absorption. In contrast, PDMAPS/NdFeBPs composites maintain a gel‐like state, facilitating reversible deformation and efficient mechano–magneto–electric energy conversion. These composites function as self‐powered tactile sensors capable of detecting strain, encoding Morse code, and operating reliably over extended cycles.

Importantly, the tunability in sol–gel transition not only governs the mechanical robustness and responsiveness of the composites but also unlocks multifunctionalities, including long‐term EMI shielding stability, dynamic strain sensing, and self‐powered signal transduction. The study highlights sol–gel modulation as a powerful strategy for engineering next‐generation soft materials and devices with adaptive electromagnetic and electromechanical functionalities.

## 4 Experimental Section

### Materials

DMAPS, ammonium persulfate (APS), DMSO (≥99.7%), DMF (anhydrous, 99.8%), ethanol (gradient grade), 1,4‐dioxane (≥99.0%), toluene (≥99.5%) were purchased from Sigma–Aldrich. Ti_3_C_2_T_x_ MXene was synthesized by selectively etching Al phase from Ti_3_AlC_2_ MAX phase, followed by delamination.^[^ ^55^ ^]^ NdFeBPs (5 µm in diameter) were purchased from Magnequench. Monodisperse PS microspheres were prepared as reported, dried under vacuum for 24 h, and stored in air.^[^ ^56^ ^]^ Ultrapure water (Direct‐Q 3 UV) was used throughout experiments.

### Preparation of PDMAPS Powder

10.06 g DMAPS and 82.14 mg APS were dispersed in 50 mL ultrapure water in a 250 mL flask. This mixture was purged with nitrogen for 10 min. Then, the mixed solution was heated at 70 °C in an oil bath at 250 rpm overnight. PDMAPS powder was recovered by water dialysis for two days and then lyophilized.

### Preparation of PDMAPS Organohydrogels

1 g of PDMAPS was dissolved in 1 mL of ultrapure water. Subsequently, 0.4 mL of DMSO was added to the 1 mL PDMAPS solution and manually stirred to induce phase separation. After 2 min of stirring, the PDMAPS organohydrogel was formed (Movie S1, Supporting Information).

PDMAPS organohydrogels with varying DMSO contents (0, 17, 29, 38, 44, and 50% v/v) and polymer concentrations (50, 56.5, and 60 wt.%) were prepared by adjusting the DMSO and polymer concentrations. PDMAPS‐based organohydrogels with different organic solvents were studied using DMSO, DMF, toluene, ethanol, and 1,4‐dioxane, with these solvents introduced at 29% (v/v). The notation v/v denotes the volume of DMSO as a fraction of the total volume of the prepared mixture.

The composition of the organohydrogels was determined by first measuring the volume and density of the residual liquid after introducing DMSO into the PDMAPS solution. The proportions of DMSO and water in the remaining liquid were calculated based on the measured densities and their corresponding values in Figure S25a (Supporting Information). The volume of DMSO retained in the organohydrogels was obtained by subtracting the residual DMSO from the initially added amount. Subsequently, the prepared organohydrogels were freeze‐dried to determine the final weight of PDMAPS. The water content in the organohydrogels was then calculated using the following equation:

(1) $$v_{water}=\frac{m_{soft\;composite}-v_{DMSO}\times \rho_{DMSO}-m_{PDMAPS}}{\rho_{water}}$$

where *v*_water_ and *v*_DMSO_ are the volumes of water and DMSO in the PDMAPS organohydrogel, respectively; *m*_soft composite_ and *m*_PDMAPS_ are the weight masses of the organohydrogel and PDMAPS in the organohydrogel, respectively; *ρ*_DMSO_ and *ρ*_water_ are the densities of the DMSO and water in the organohydrogel, respectively.

The calculated compositions of DMSO (volume), water (volume), and PDMAPS (weight mass) in an organohydrogels prepared with 29% v/v DMSO is shown in Figure S25b (Supporting Information).

### Preparation of PDMAPS‐Based Soft Composites

PDMAPS/MXene composites were synthesized by incorporating MXene into the PDMAPS solution at concentrations of 1, 2, 3, and 4 wt.%, followed by the addition of DMSO to form the final composites. The accurate MXene content in the composite was determined using both thermogravimetric analysis (TGA) and direct weighing (using the 4 wt.% MXene sample as an example). For TGA, the lyophilized PDMAPS/MXene composite (with solvent removed) was analyzed up to 800 °C, yielding a residual mass of 7.62 wt.% (Figure S26, Supporting Information). After correcting for solvent removal, the estimated MXene content was ≈5.94 wt.%. To further verify this value, direct weighing was performed on the hydrated composite. Since the removed solvent was nearly transparent (Figure S27, Supporting Information)—indicating negligible MXene residue—the MXene weight fraction was calculated to be 4.52 ± 0.61 wt.% (mean ± SD from *n* = 3 independent samples), based on the total mass of the soft composite and the known initial MXene input mass.

PDMAPS/NdFeBPs soft composites were fabricated by adding NdFeBPs into the PDMAPS solution at 9, 17, 23, 29, and 33 wt.%, and DMSO was added to obtain the magnetic soft composites.

PDMAPS/PS composites were synthesized by incorporating PS into the PDMAPS solution at concentrations of 5 wt.%, followed by the addition of DMSO to form the final composites.

### Preparation of Self‐Powered Device

The self‐powdered model was synthesized using poly(2‐hydroxyethyl methacrylate) (PHEMA) gel, prepared in accordance with relevant literature.^[^ ^20^ ^]^ The model was shaped into a hollow cylinder with an outer diameter of 15 mm, an inner diameter of 7 mm, and an internal height of 10 mm. A stainless‐steel spring, configured as a 7‐layer spiral with a thickness of 0.5 mm, a diameter of 6 mm, and a height of 10 mm, was placed at the center of the hollow cylindrical model. The well‐prepared PDMAPS/NdFeBPs composite (33 wt.% NdFeBPs) was carefully squeezed into the hollow model to form the self‐powered device. The samples were magnetized using a variable gap magnet (EM‐8641, PASCO), with a magnetization distance of 5 mm between the sample and the two magnets. The mold for the preparation of the PHEMA gel and the holder used for magnetization were 3D printed using Ultimaker S3.

### Analytical Techniques

FTIR spectrum were recorded by PerkinElmer FTIR with attenuated total reflection (ATR). ^1^H NMR was recorded by a Bruker Avance III 400 MHz NMR spectrometer (AVIII400). The microstructure images of samples were monitored using a scanning electron microscope (FE‐SEM, Sigma VP, Zeiss) observing an SE2 pattern. The tensile strength and compressive strength of samples were performed by an Instron 5567 materials testing system at room temperature. TGA was performed using a TGA 5500 thermal analyzer, with a heating rate of 10 °C min^−1^ from 30 °C to 800 °C under a nitrogen atmosphere.

### Rheology Measurement

All rheological measurements were taken on a rheometer (Anton Paar MCR302) with a 25 mm parallel plate at 298 K, using a fixed gap of 1 mm. Dynamic strain sweep measurements from 0.01% to 100% were performed at the frequency of 10 rad s^−1^. Dynamic frequency sweep measurements from 0.1 to 100 rad s^−1^ were performed with the shear strain of 1%.

### Electrical Measurements

Resistance signals were measured on a Keithley 2634B system source meter at a direct voltage of 0.1 V. The soft composite was sandwiched between two conductive thin copper plates connected to conductive wires. The contact area between the hydrogel and copper plates was ≈50 mm^2^, with a thickness of ≈5 mm. The conductivity *σ* of the sample can be calculated according to the following equation:

(2) $$\sigma =\frac{l}{RA}\;$$

where, *l* is the thickness of the composite, *R* the electrical resistance of the composite, *A* the contact area between the composite and copper plates.

### DFT Calculations

The binding energies between DMAPS and water and different solvents (DMSO, ethanol, DMF, toluene, and 1,4‐dioxane) were calculate with Gaussian 16 using the ωB97XD/6‐311G (d,p) DFT method.^[^ ^57^, ^58^ ^]^ Geometry optimizations were performed. The solvation effect was using the integral equation formalism variant (IEFPCM) method with all solvents except 1,4‐dioxane and DMF as default solvents.^[^ ^59^ ^]^ The dielectric constants of 1,4‐dioxane and DMF were defined separately.

### EMI Shielding Measurements

EMI shielding capacity measurements were performed in the frequency range between 5.38 and 8.17 GHz by a coaxial air‐line method using a microwave network analyzer (ROHDE & SCHWARZ ZNA67). The EMI shielding samples were prepared using a custom polylactic acid (PLA) mold fabricated by 3D printing (Ultimaker S3). The mold's internal cavity measured 2 cm × 4 cm with an initial thickness of 1 mm. For strain‐dependent EMI measurements, a predetermined mass of the PDMAPS/MXene composite was introduced into the mold to achieve a thickness of 1 mm. This mass was maintained across all subsequent tests under different compressive strains to ensure consistency. Scattering parameters were obtained to calculate the EMI shielding effectiveness (SE) and the power coefficients. The power coefficients for reflection (*R*), adsorption (*A*) and transmission (*T*) can be calculated as follows:^[^ ^60^ ^]^

(3) $$R={\left|S_{11}\right|}^{2}={\left|S_{22}\right|}^{2}$$

(4) $$T={\left|S_{12}\right|}^{2}={\left|S_{21}\right|}^{2}$$

(5) A=1−R−T

The effective absorptance (*A_eff_*) can be expressed as:

(6) $$A_{eff}=\frac{1-R-T}{1-R}\;$$

The detailed calculations of the total EMI SE (SE_Total_) and its absorption (SE_Absorption_) and reflection (SE_Reflection_) components can be described as:

(7) $$SE_{Total}=SE_{Reflection}+SE_{Absorption}$$

(8) $$SE_{Reflection}=10\log\frac{1}{1-R}=10\log\frac{1}{1-{\left|S_{11}\right|}^{2}}$$

(9) $$SE_{Absorption}=10\log\frac{1}{1-A_{eff}}=10\log\frac{1-{\left|S_{11}\right|}^{2}}{{\left|S_{21}\right|}^{2}}$$

(10) $$SE_{Total}=10\log\frac{1}{T}=10\log\frac{1}{{\left|S_{21}\right|}^{2}}$$

### SAXS/WAXS Measurement

SAXS/WAXS data was obtained using a Xenocs Xeuss 3.0 SAXS/WAXS system (Xenocs SAS, Grenoble, France). The system includes a microfocus X‐ray source (sealed tube) with a Cu target and a multilayer mirror which yields a parallel beam with a nominal wavelength of 1.542 Å (combined Cu K‐α1 and Cu K‐α2 characteristic radiation). The source operates at 50 kV and 0.6 mA. The beam is collimated by a set of variable slits and the beam size at the sample was 0.4 or 0.7 mm during the experiment. The system does not include a beam stop, which enables direct measurement of sample transmission. The background scattering from sample holder is normalized and subtracted from the data according to sample transmission. The data is acquired using an area detector (Eiger2 R 1 m, Dectris AG, Switzerland) that was in the evacuated chamber. The sample‐to‐detector distance was calibrated by measuring the diffraction from a known LaB6 standard sample. The sample‐to‐detector distance for SAXS and WAXS measurements are 1100 and 55 mm respectively.

MXene‐PDMAPS samples before and after compressing (40% using a mold) was freeze dried, resulting in a spherical and a cake‐like piece, respectively. After freeze‐drying, the doll was taking out from the mold, and a thin slice sample ≈0.5 mm thickness was cutter from the pieces as show in Figure S19a (Supporting Information). The X‐ray of SAXS/WAXS measurement was perpendicular to the slices. Figure S19b,c (Supporting Information) are the 2D SAXS/WAXS data of slice samples from doll before and after compressing, respectively.

### Magnetic Moment Measurement

Magnetic measurements were performed using a Quantum Design PPMS DynaCool with samples sealed in a VSM powder sample holder (P125E) at 295 K.

### Statistical Analysis

The software Microsoft Excel for Windows (Microsoft Office, 2020) was used for statistical calculations. All results were expressed as mean ±SD (*n* = 3 independent samples).

## Conflict of Interest

The authors declare no conflict of interest.

## Author Contributions

B.P. and Z.‐P.L. conceived the experimental idea. Z.M. conducted experiments and characterizations. B.P. performed the density functional theory calculations and Z.M. analyzed the data. X.H. prepared MXene, performed electromagnetic shielding measurements and data analysis. Z.‐P.L. and X.H. performed SAXS/WAXS measurement and data analysis. Z.M. wrote the first version. All authors discussed the results and contributed to the final manuscript.

## Supporting information



Supporting Information

Supplemental Movie 1

Supplemental Movie 2

## Data Availability

The data that support the findings of this study are available from the corresponding author upon reasonable request.
