# Supplementary material for: Polyzwitterionic Organohydrogel and Soft Composite with Tunable Sol–Gel Properties Enabling On‐Demand Functionalization with Colloids
Source: Adv Sci (Weinh). 2025 Dec 14;13(12):e18531. doi: 10.1002/advs.202518531 (PMC12948205; doi:10.1002/advs.202518531)
Supplement: Supplementary file 1 — Supporting Information [file ADVS-13-e18531-s003.docx]

Supporting Information

**Polyzwitterionic Organohydrogel and Soft Composite with Tunable Sol–gel Properties Enabling On-Demand Functionalization with Colloids**

Ziyue Miao, Xiaodan Hong, Olli Ikkala,* Zhong-Peng Lv,* Bo Peng*

**Table S1.** Detailed comparison of the EMI shielding performance and key features of MXene-based hydrogels and organohydrogels ("--" denotes data not available or not mentioned).

| **Materials** | **Thickness (mm)** | **SE_Tmax_ (dB)** | **Stability (1 week)** | **Strain-tunable EMI** | **Versatility** | **Ref.** |
| --- | --- | --- | --- | --- | --- | --- |
| Mxene/PDMAPS | 1 | 18.76 | High (98.40% of original SE_T_) | Yes | EMI shielding, electromechanical–magneto–electric conversion | **This work** |
| MXene/PEDOT:PSS/PVA | 4.5 | 39 | -- | Yes | EMI shielding, strain sensing | [1] |
| MXene/PVA/AAM | 3.8 | 32.8 | 77.10% | Yes | EMI shielding, electrical response | [2] |
| MXene/PAA/CS | 1 | 18.76 | 57.05% | -- | EMI shielding, strain sensing | [3] |
| MXene/PVA/AgNWs | 1 | 31 | -- | -- | EMI shielding, strain sensing | [4] |
| MXene/PNIPAM | 2 | 59.3 | -- | -- | EMI shielding, temperature response and optical response | [5] |
| MXene/PVA | 2 | 57 | -- | -- | EMI shielding, strain sensing | [6] |


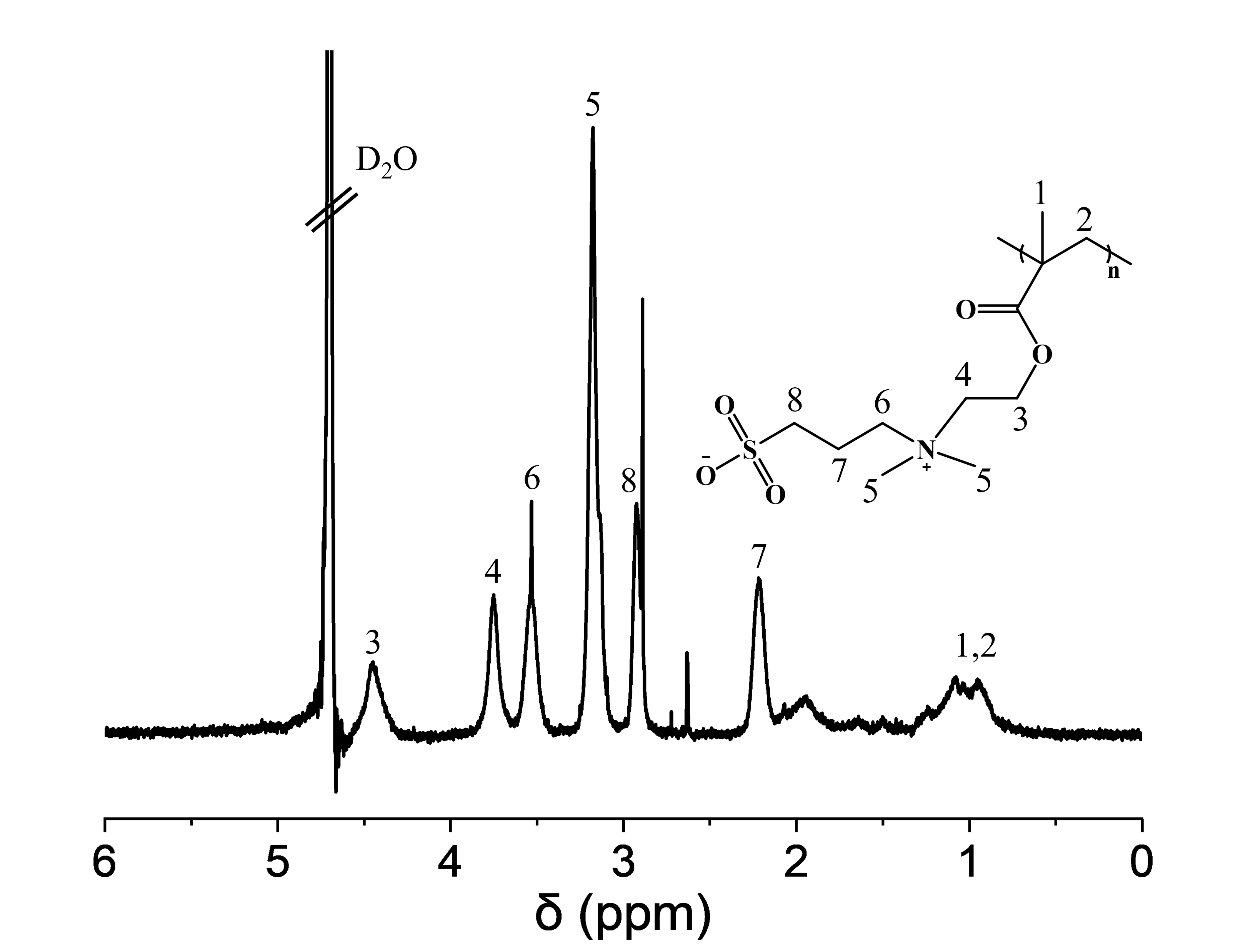


**Figure S1.** ^1^H NMR spectra of PDMAPS in D_2_O.


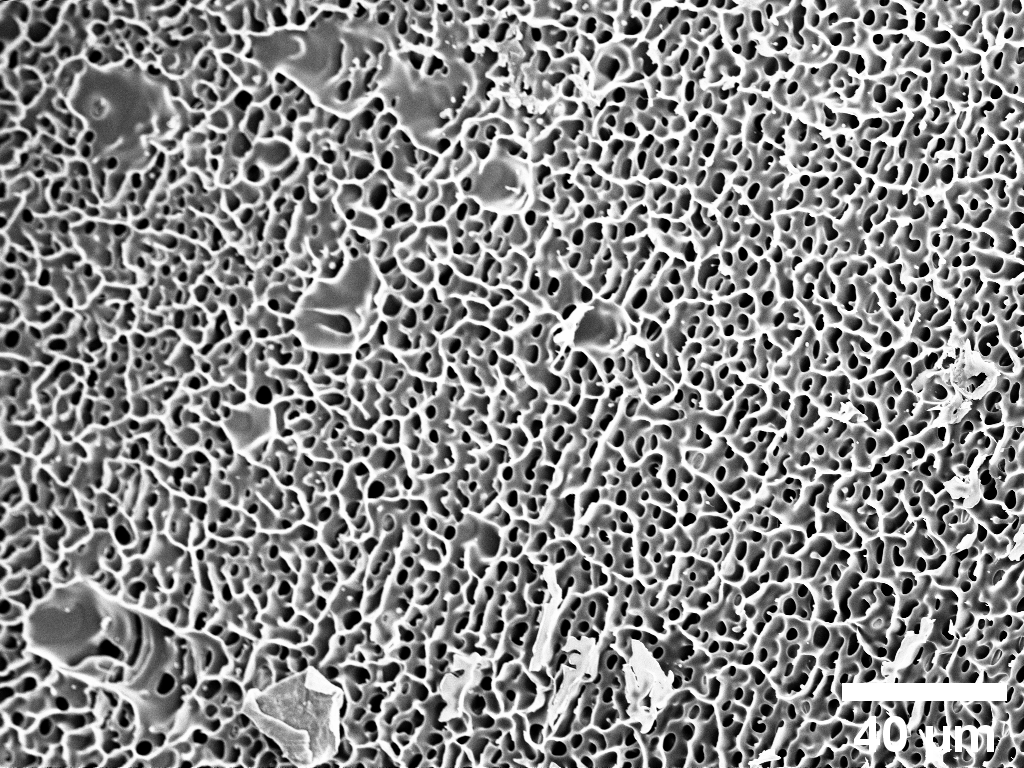


**Figure S2.** SEM image of freeze-dried PDMAPS powder obtained after polymerization in aqueous solution, dialysis, and lyophilization. The powder was redissolved in water (50 wt.%) before freeze-drying for SEM observation, showing a porous morphology due to the highly hydrated and loosely associated polymer chains.


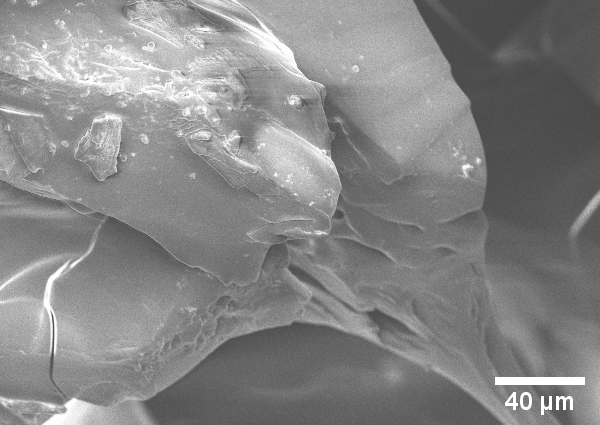


**Figure S3.** SEM image of the freeze-dried PDMAPS organohydrogel (29 % v/v DMSO). DMSO enhances ionic associations between sulfonate and ammonium groups, leading to polymer domain densification and a compact morphology.


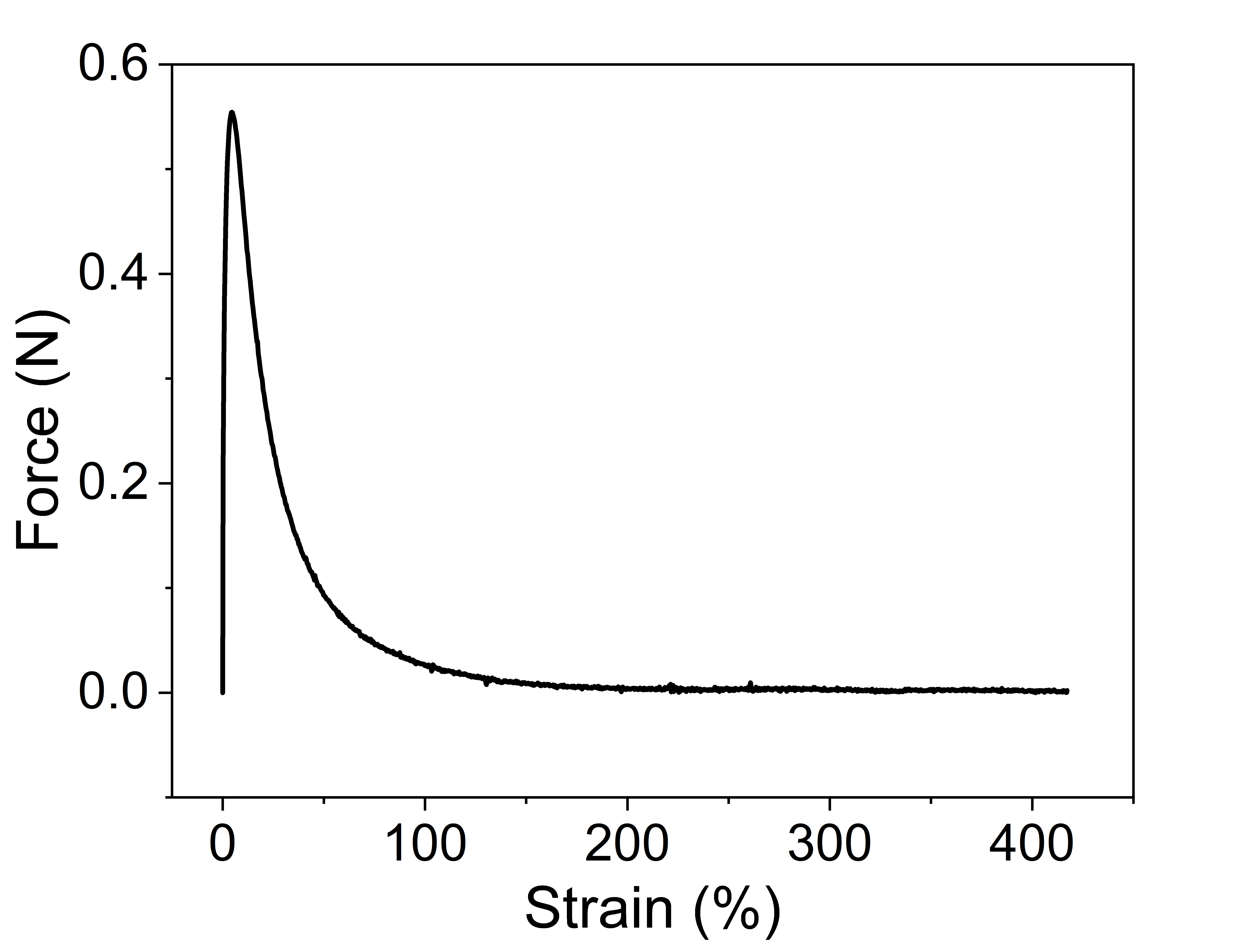


**Figure S4.** Tensile stress-strain curve of the PDMAPS organohydrogel.


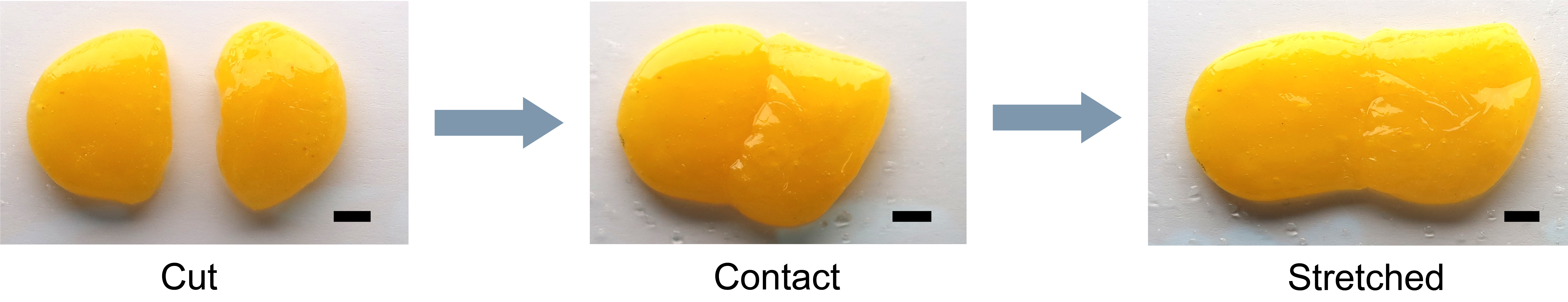


**Figure S5.** Demonstration of self-healing ability (dyed by quinoline yellow). Scale bar 0.5 cm.


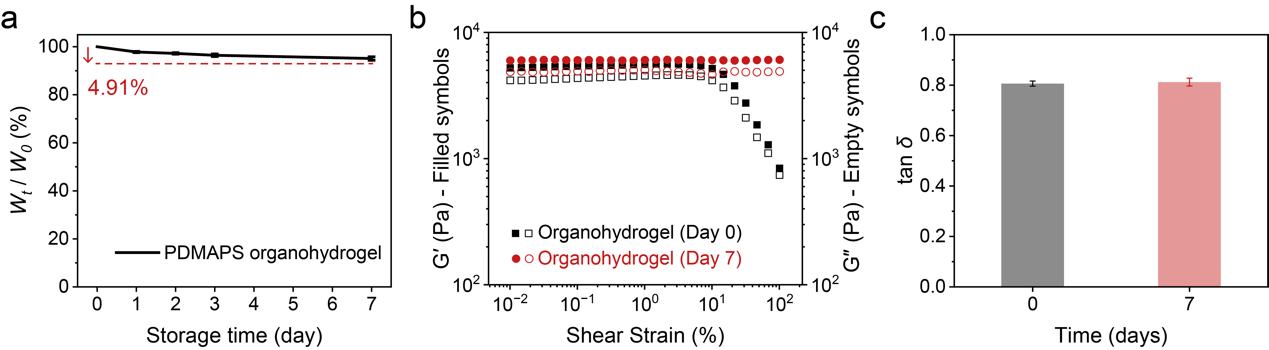


**Figure S6.** Long-term stability of the PDMAPS organohydrogel (29 % v/v DMSO). a) Mass content retention over 7 days (sample size *n* = 3, error bars correspond to standard deviation, SD). b) Strain amplitude sweep curves of the organohydrogel at Day 0 and Day 7. c) tan δ of the organohydrogel at Day 0 and Day 7, determined within the linear viscoelastic region from (b).


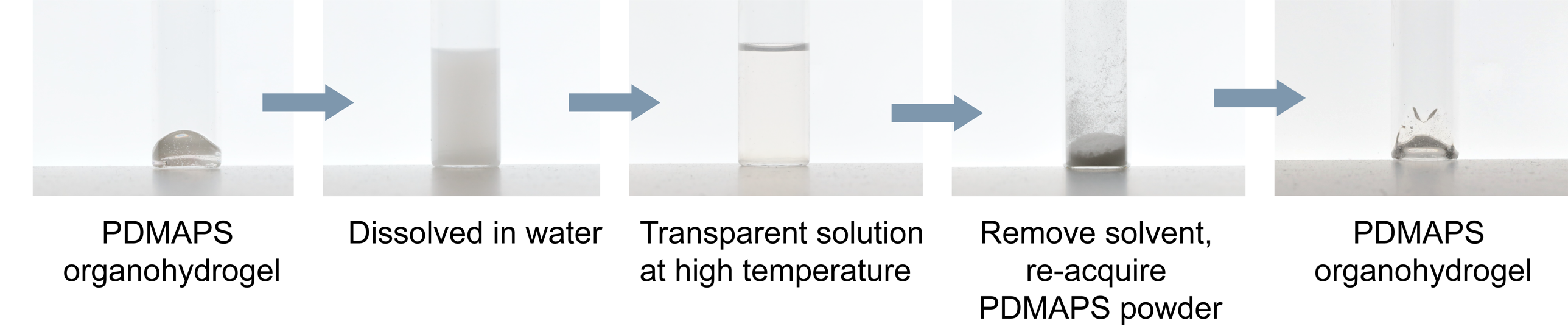


**Figure S7.** Reusability of PDMAPS organohydrogel.


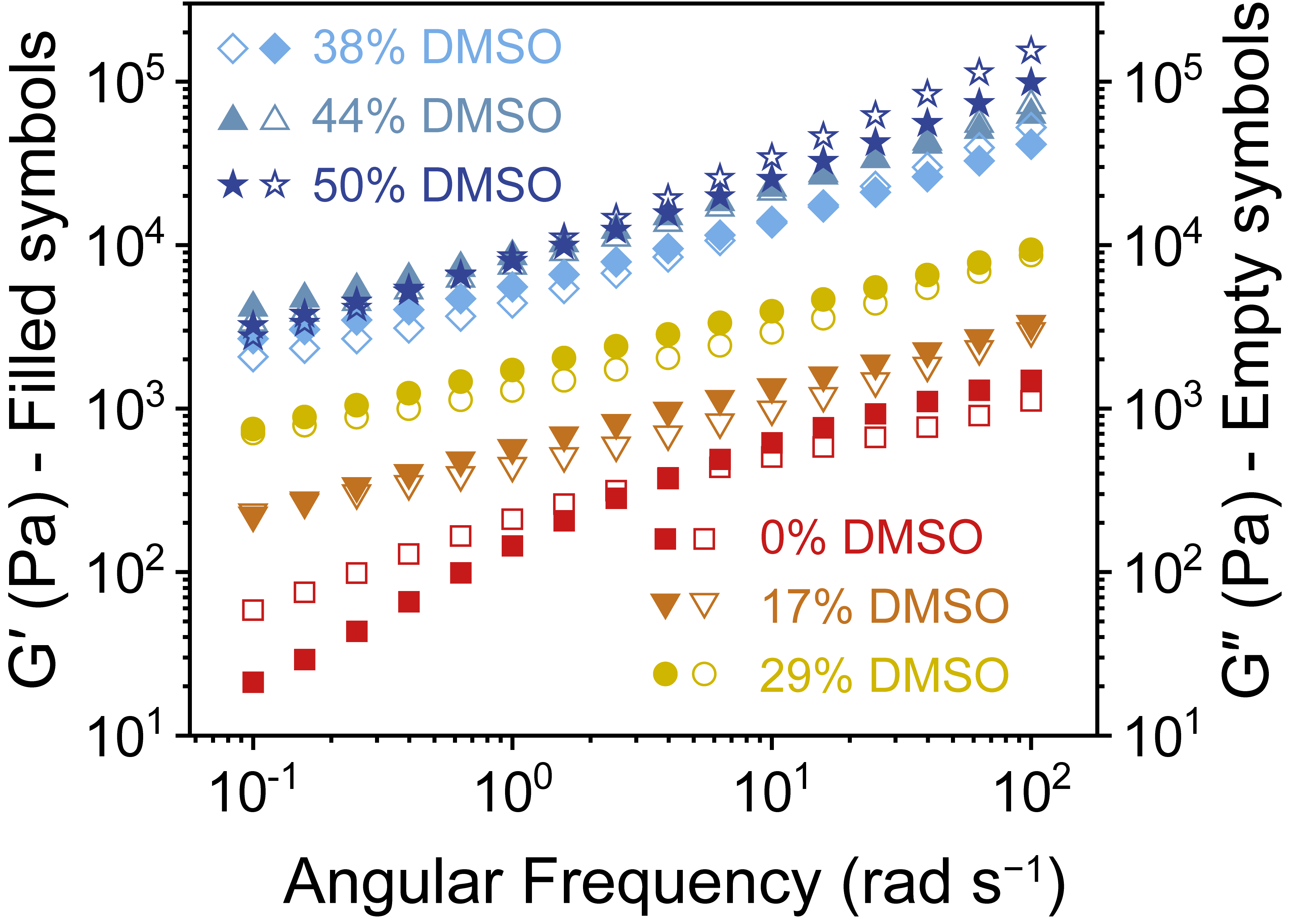


**Figure S8.** Frequency sweeps of PDMAPS organohydrogel with different DMSO contents (% v/v, relative to the total volume of the prepared mixture).


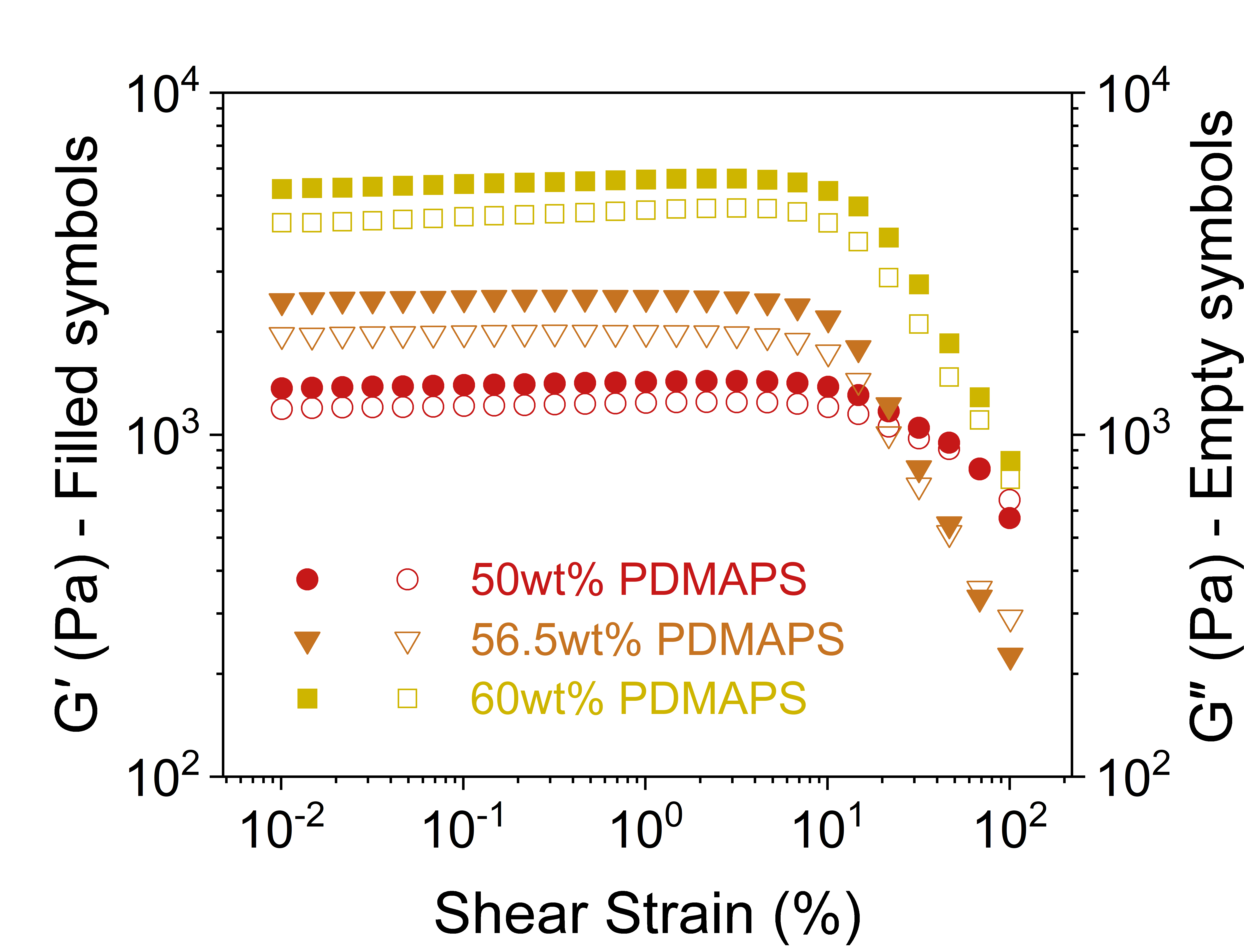


**Figure S9.** Strain amplitude sweeps of PDMAPS organohydrogel with different PDMAPS concentrations (wt.%).


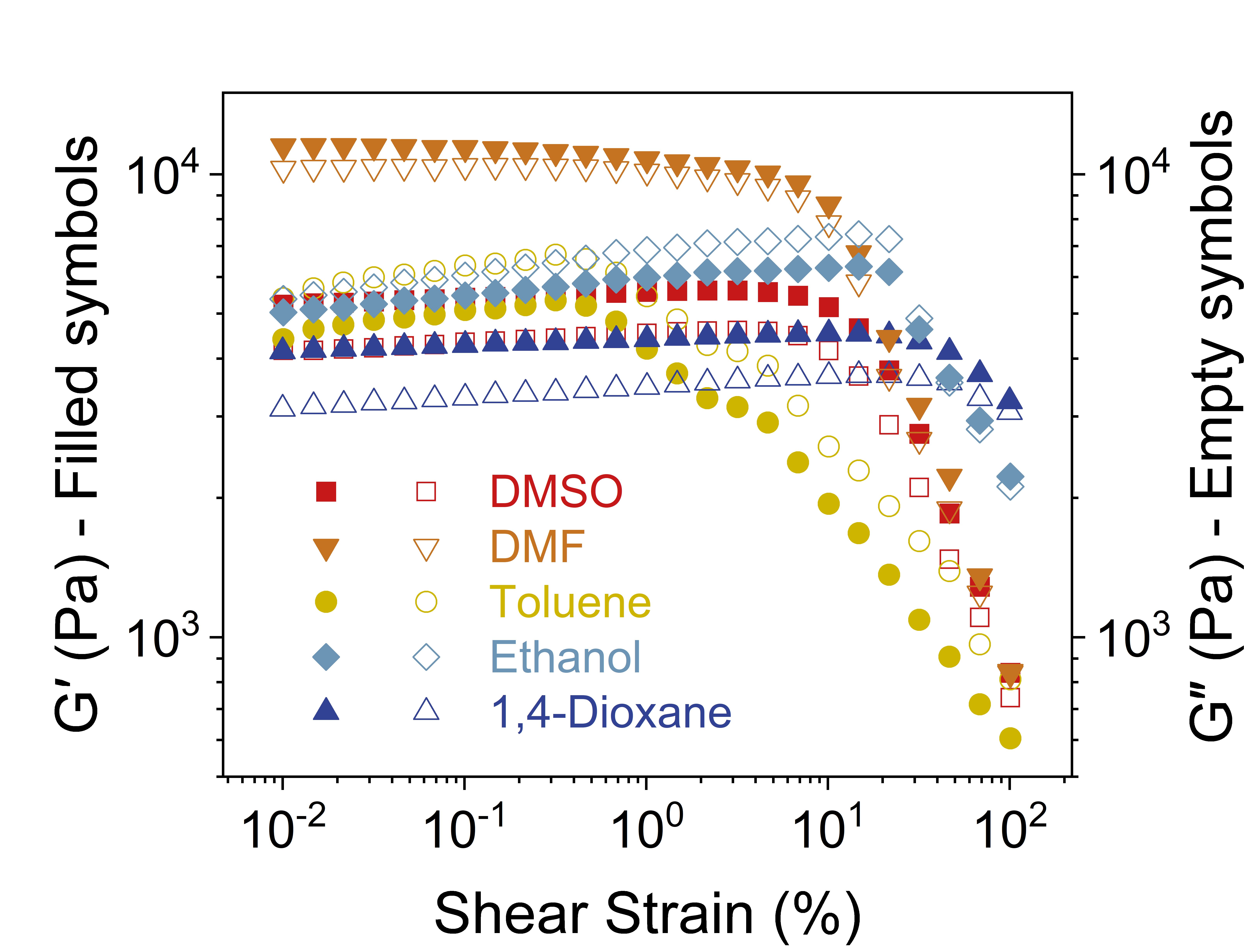


**Figure S10.** Strain amplitude sweeps of PDMAPS organohydrogels with different organic solvents.


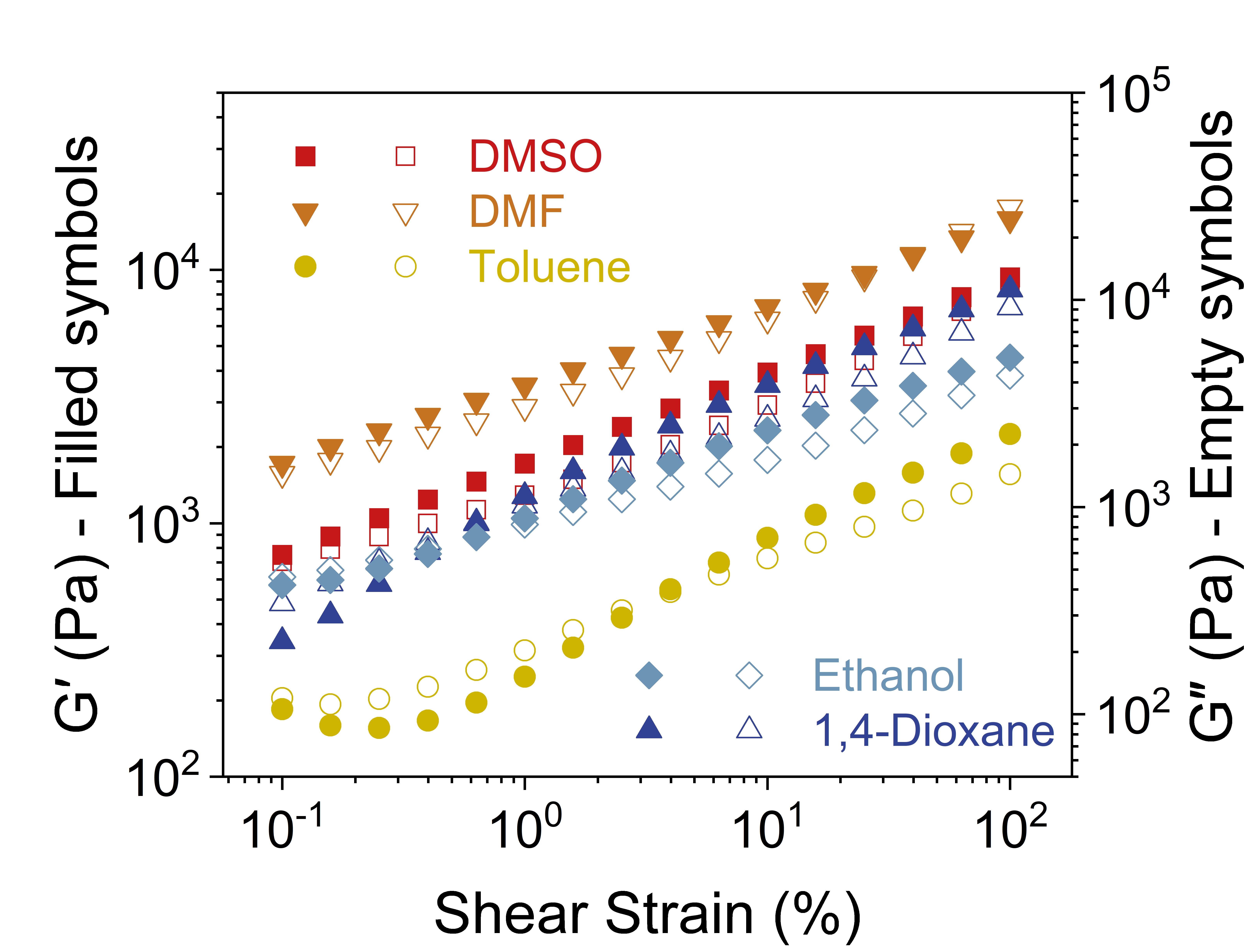


**Figure S11.** Frequency sweeps of PDMAPS organohydrogels with different organic solvents.


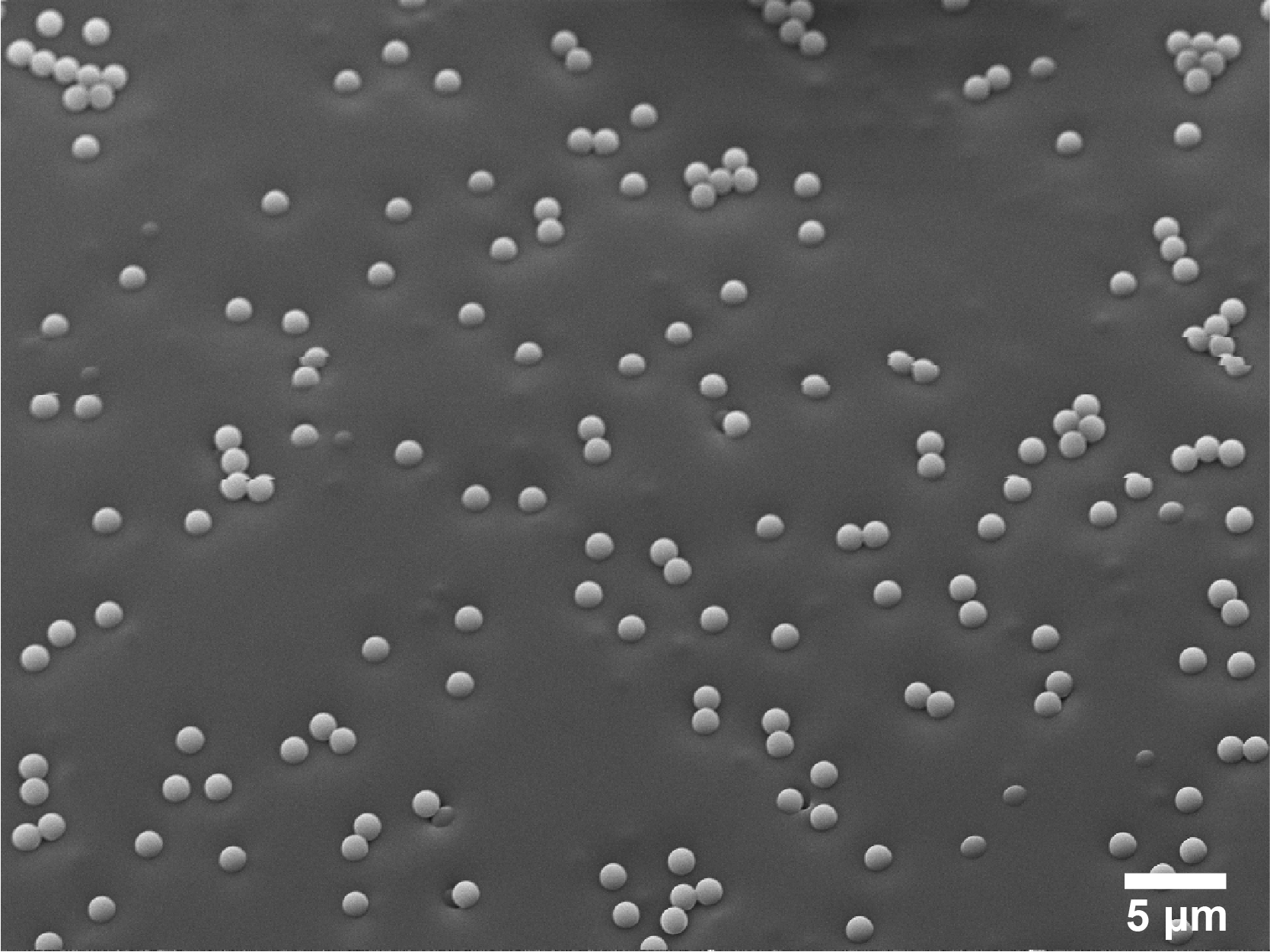


**Figure S12** SEM image of the freeze-dried PDMAPS/polystyrene (PS) composite showing uniformly dispersed PS microspheres, indicating homogeneous filler distribution within the polymer matrix.


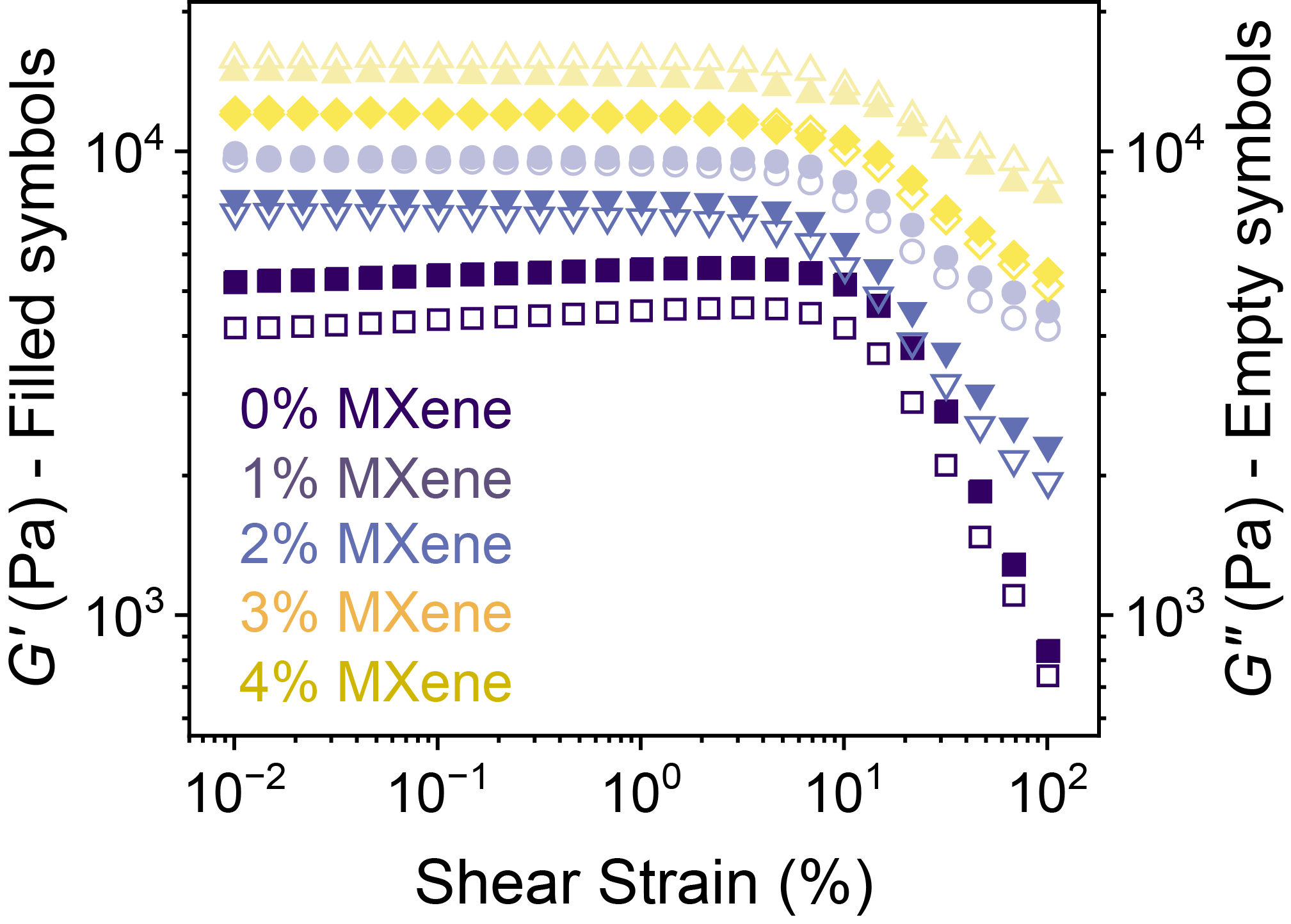


**Figure S13.** Strain amplitude sweeps of samples with different MXene contents (wt.%).


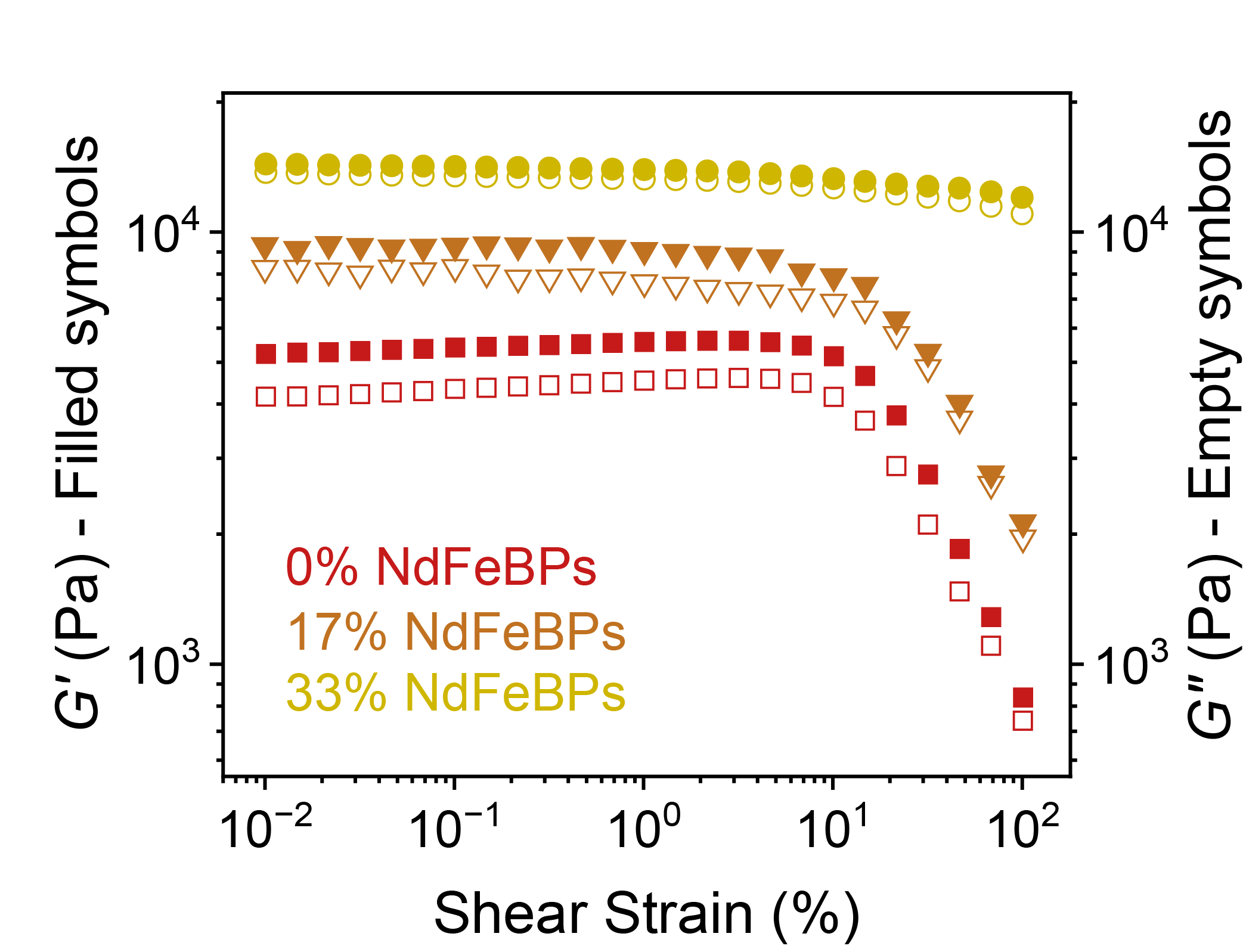


**Figure S14.** Strain amplitude sweeps of PDMAPS/NdFeBPs soft composites with different NdFeBPs contents (wt.%).


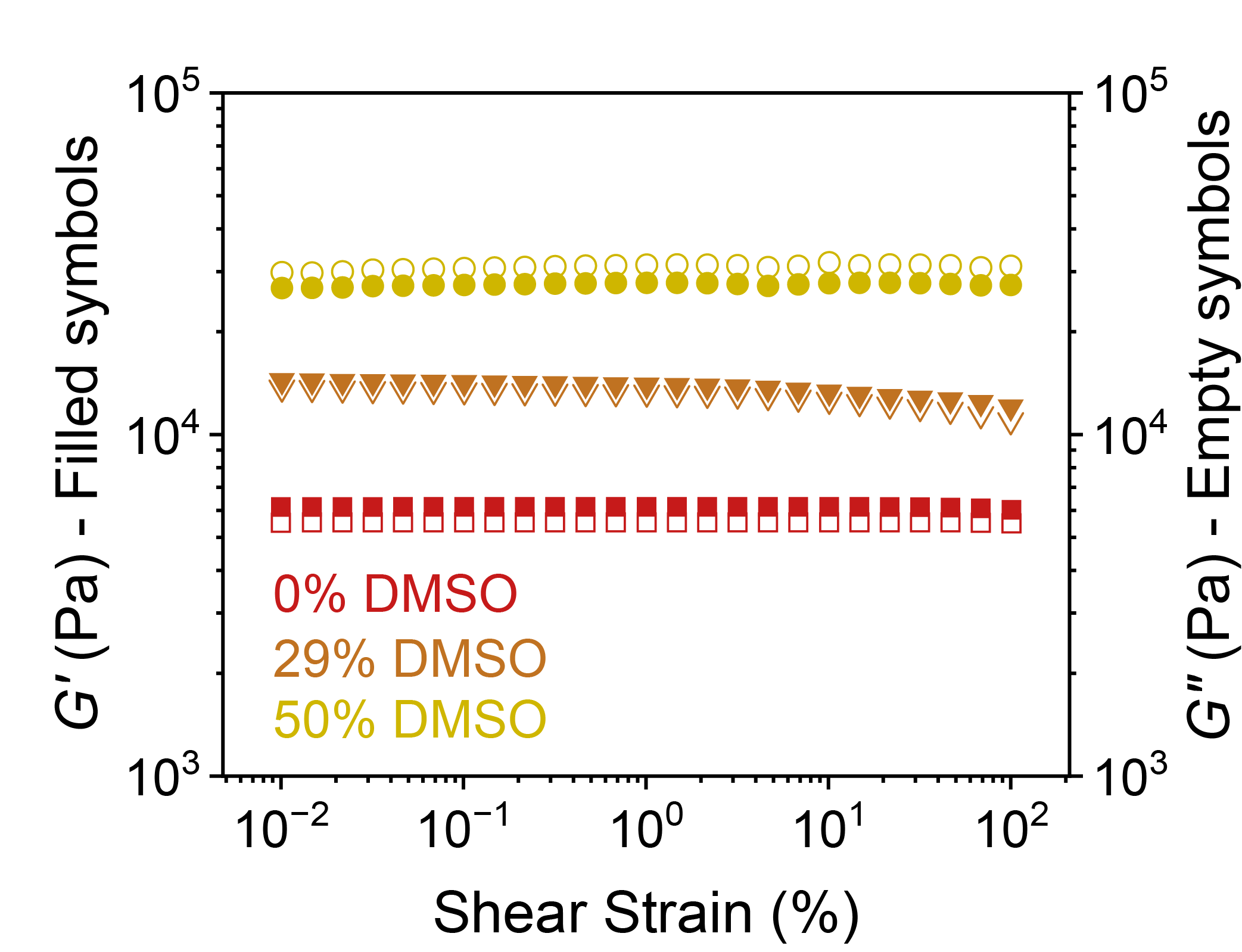


**Figure S15.** Strain amplitude sweeps of PDMAPS/NdFeBPs soft composites with different DMSO contents (% v/v, relative to the total volume of the prepared mixture).


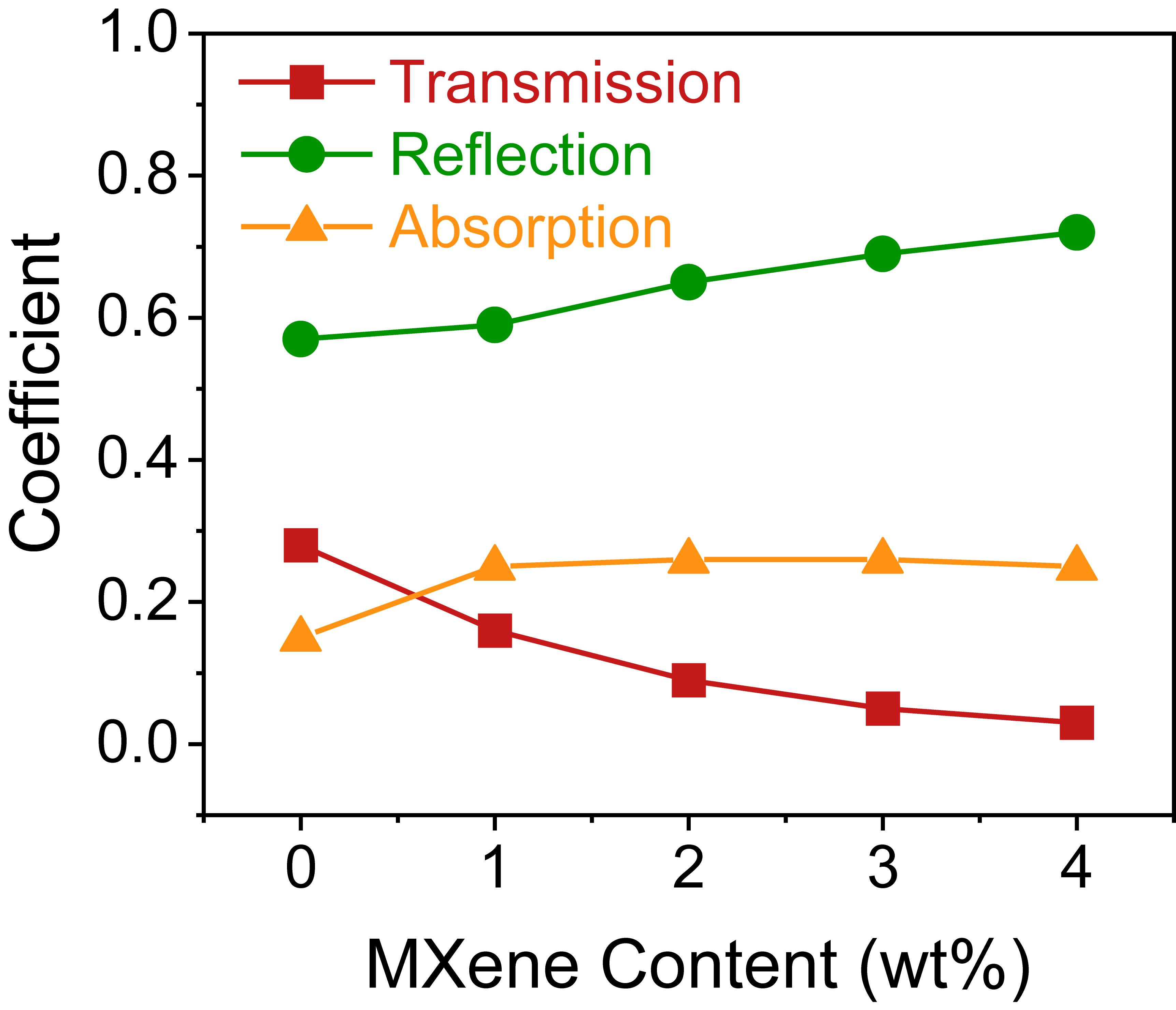


**Figure S16.** Power coefficients of transmission, reflection, absorption of PDMAPS/MXene soft composites with different MXene contents (wt.%).


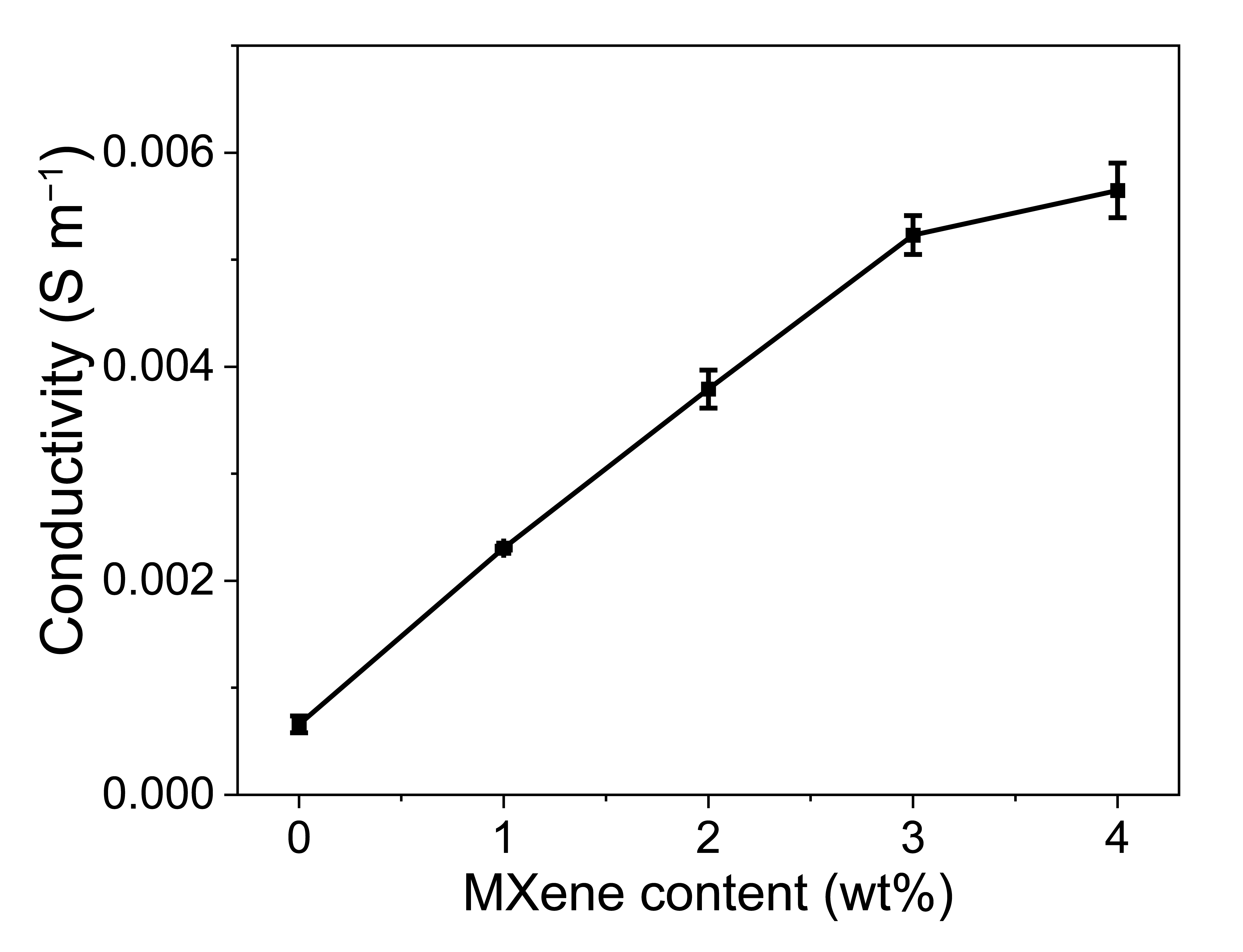


**Figure S17.** Conductivity of PDMAPS/MXene composites with different MXene contents. Sample size *n* = 3; error bars correspond to SD.


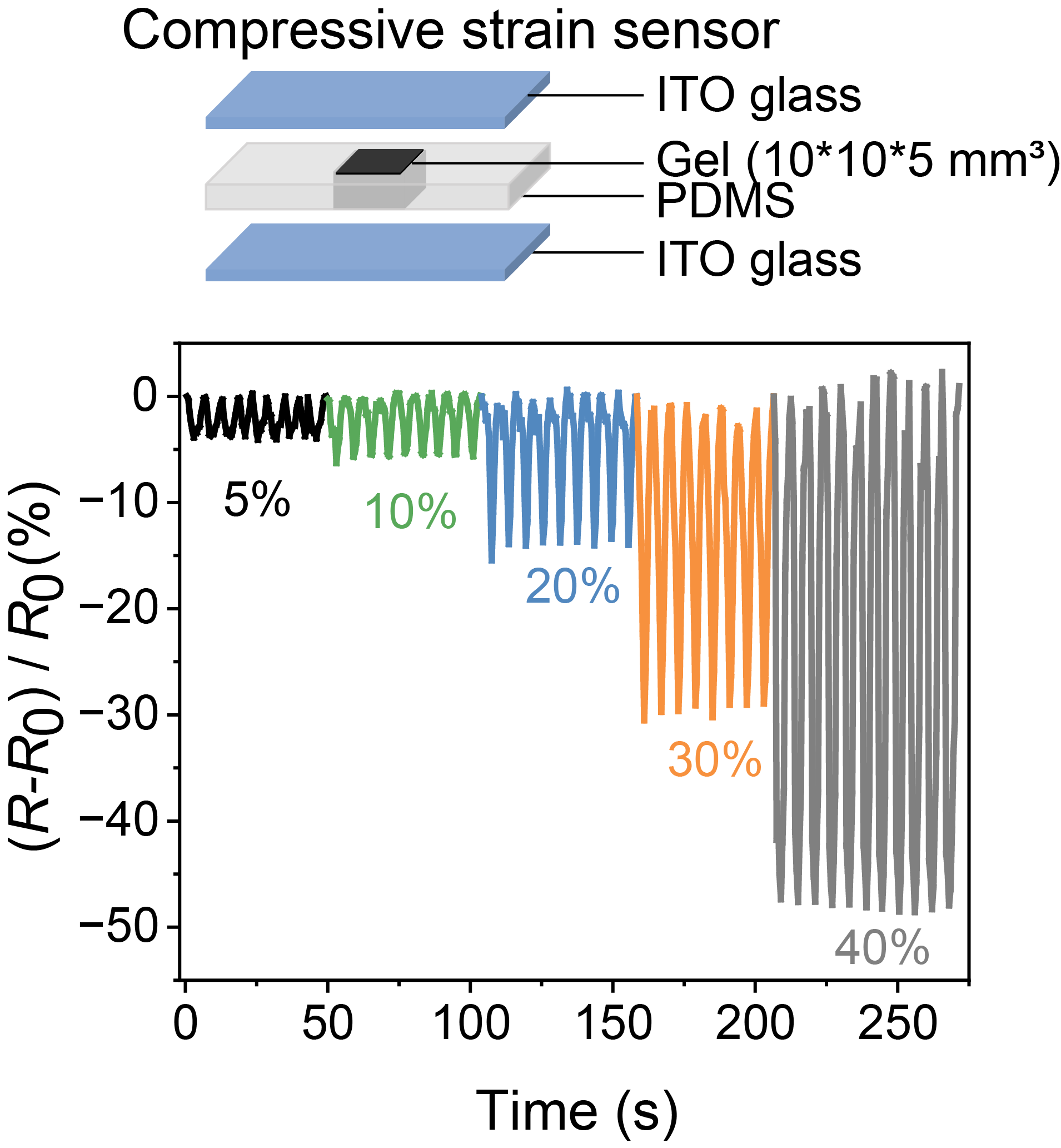


**Figure S18.** Demonstration of the PDMAPS/MXene organohydrogel–based strain sensor. Relative resistance changes ((*R*-*R*_0_)/*R*_0_) under different compressive strains at a rate of 5 mm min^-1^.


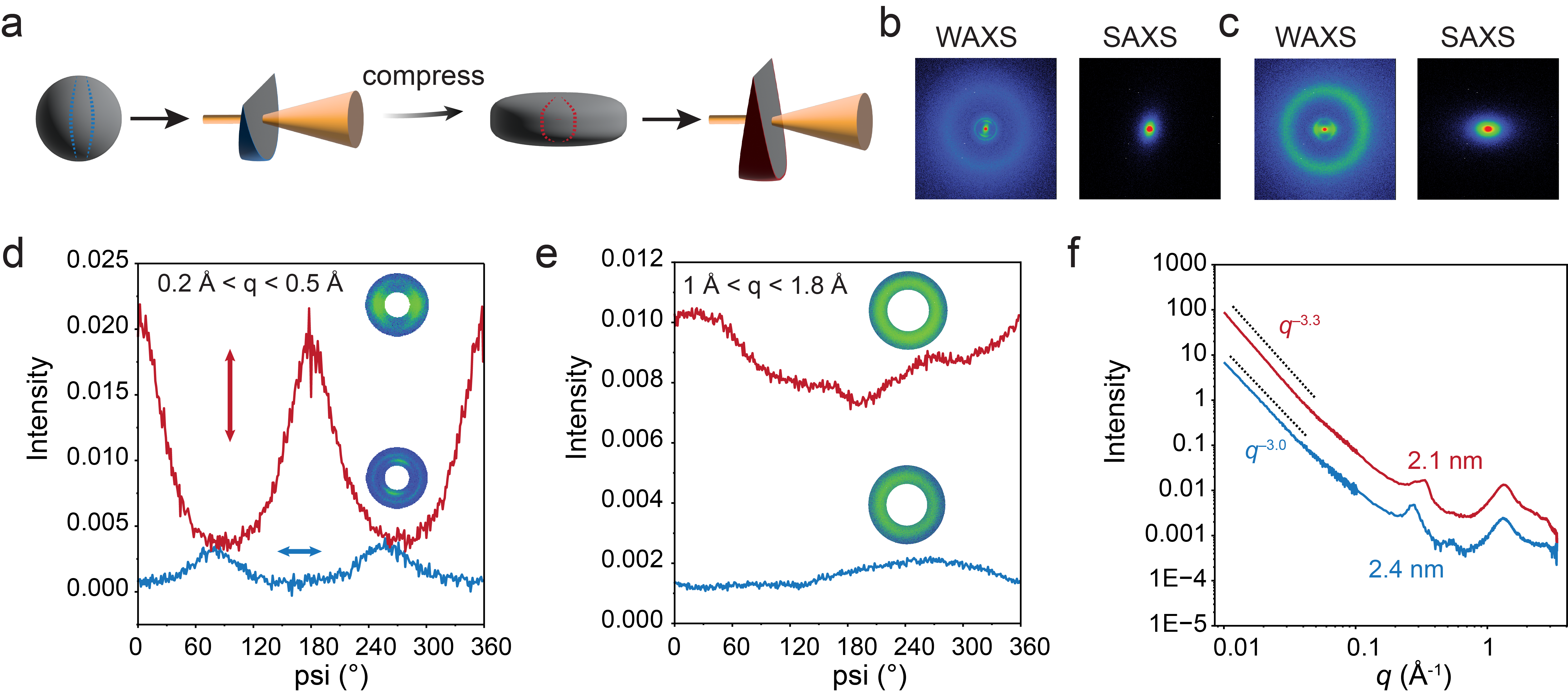


**Figure S19.** Ex-situ SAXS/WAXS characterization of PDMAPS/MXene composites before and after 40% compression. (a) Sample preparation schematic showing slice extraction for SAXS/WAXS analysis. The X-ray was perpendicular to the slices. (b,c) 2D SAXS/WAXS patterns of uncompressed and compressed samples, respectively. (d) Azimuthal intensity profiles of 0% (blue) and 40% (red) samples in the *q* range between 0.2 Å^−1^ and 0.5 Å^−1^, corresponding to the (002) peak of MXene lamella structures. (e) Azimuthal intensity profiles of 0% (blue) and 40% (red) samples in the *q* range between 1 Å^−1^ and 1.8 Å^−1^, corresponding to the atomic order in PDMAPs matrix. (f) Comparison of *d*-spacing and Porod slopes indicating enhanced nanosheet alignment and smoother MXene–polymer interfaces after compression.


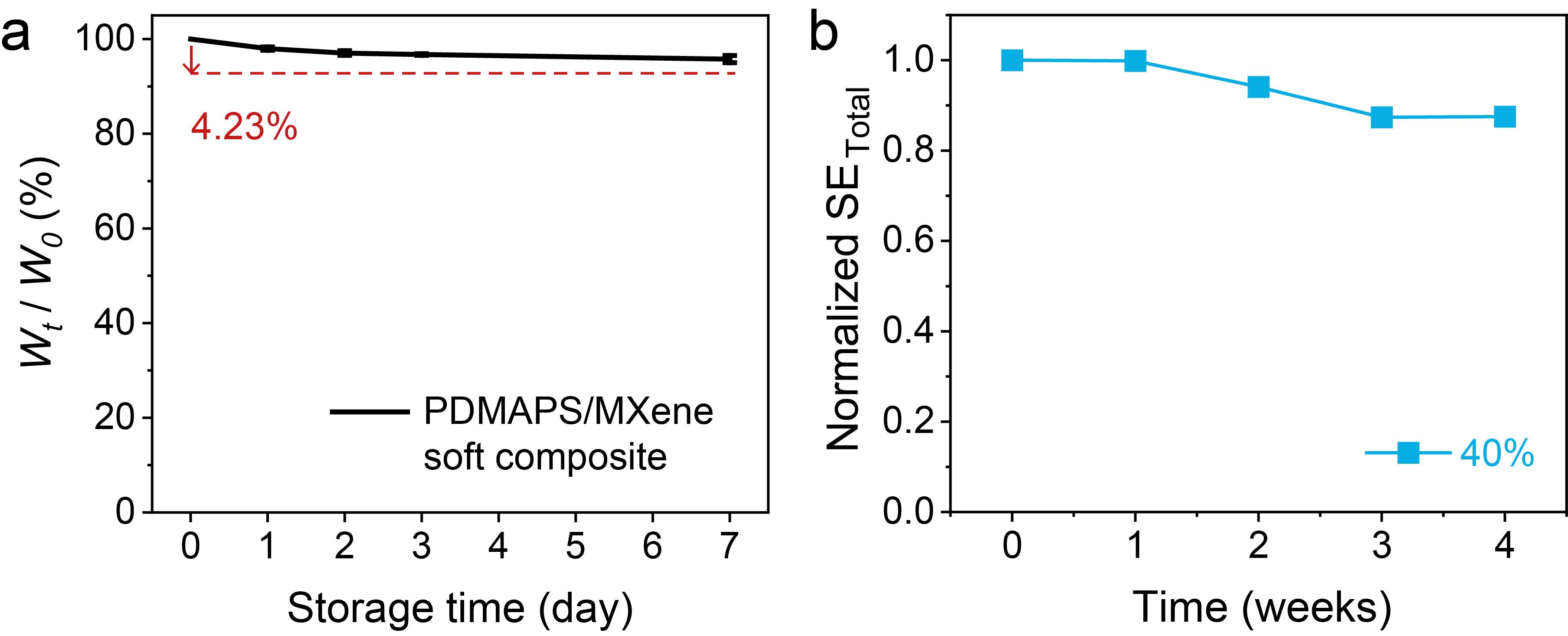


**Figure S20.** Long-term stability of the PDMAPS/MXene soft composite. a) Mass content retention over 7 days (*n* = 3; error bars represent the SD). b) Time-dependent stability of PDMAPS/MXene at 40% compressive strain.


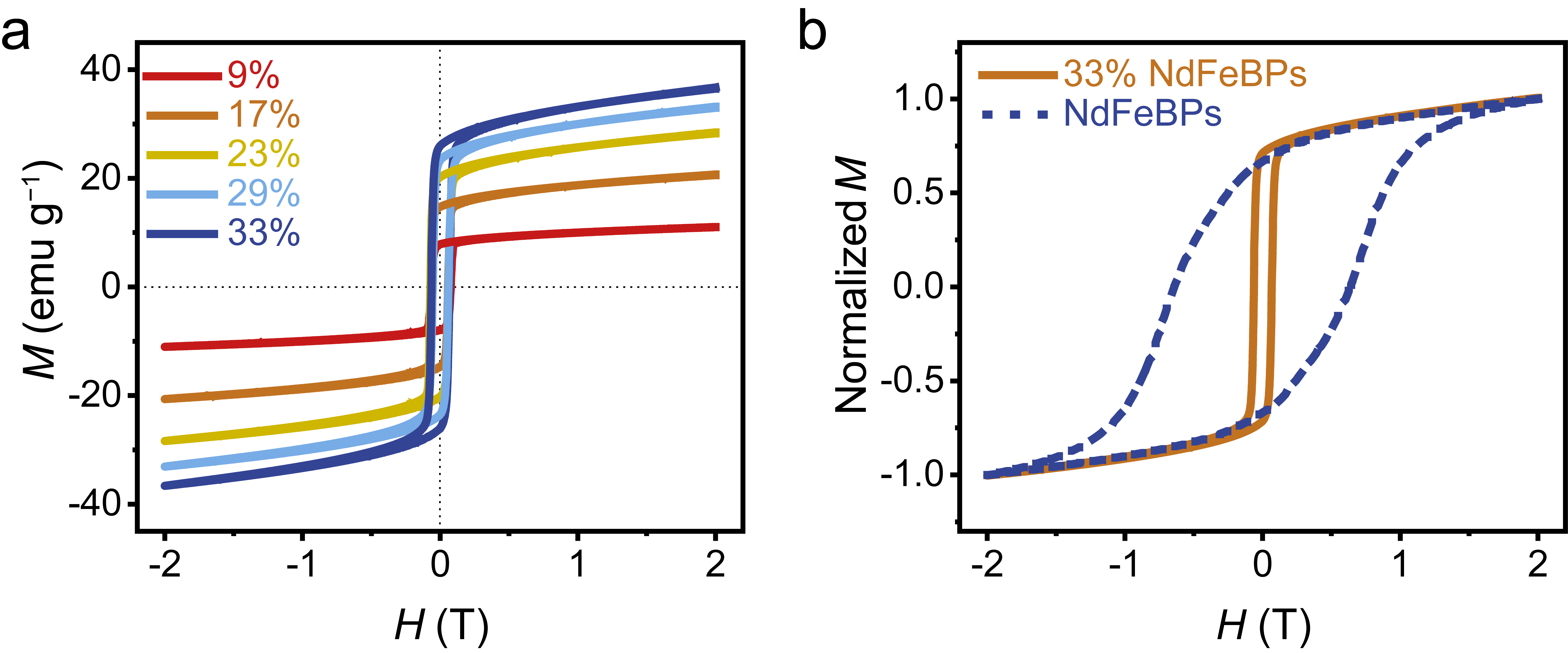


**Figure S21.** a) Hysteresis loops of magnetic PDMAPS/NdFeBPs soft composites with different NdFeBPs contents (wt.%). b) Normalized magnetization of NdFeBPs particles and PDMAPS/NdFeBPs magnetic composites (33 wt.% NdFeBPs).


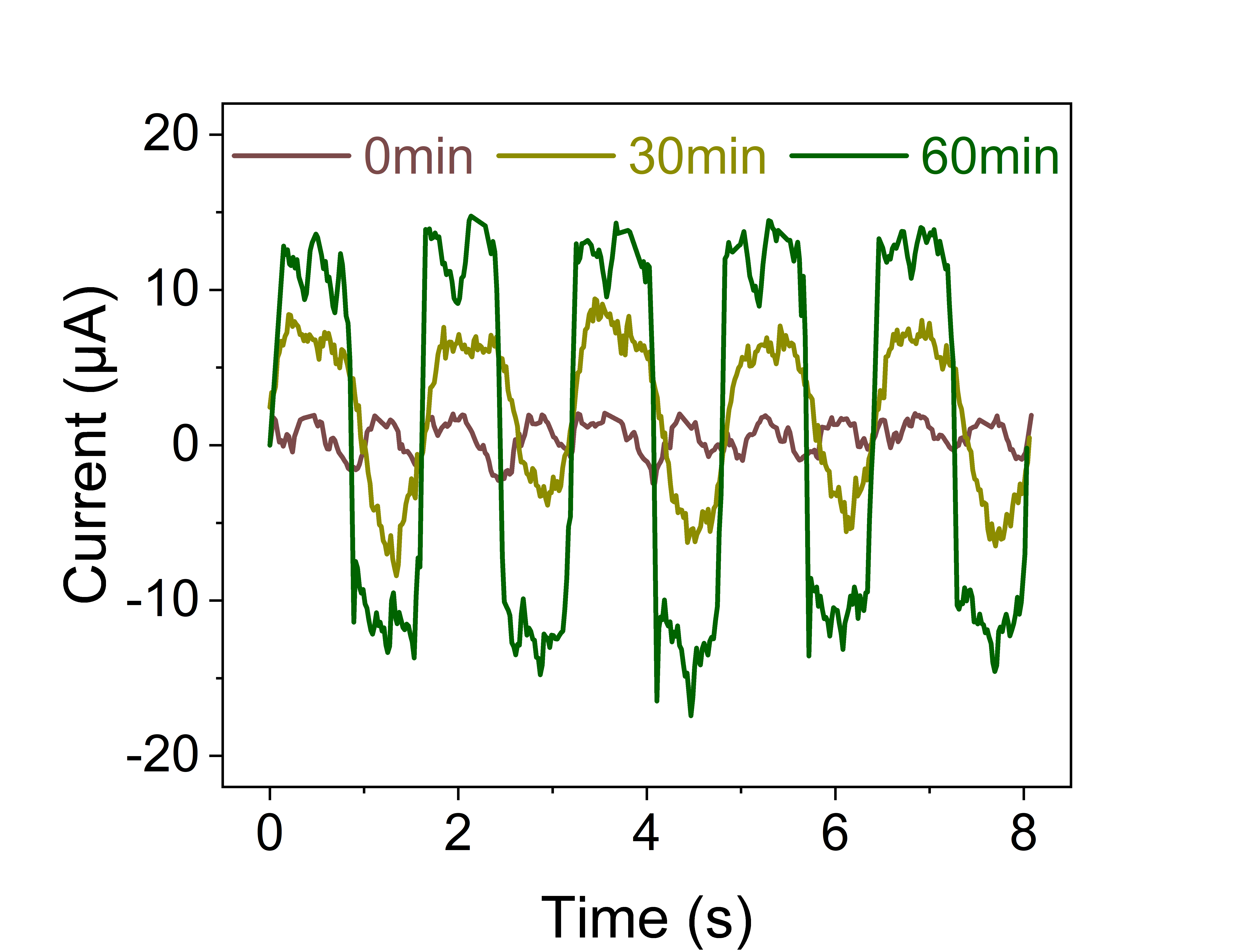


**Figure S22.** Output current plots of the magnetoelectric device with PDMAPS/NdFeBPs soft composites (33 wt.% NdFeBPs) after 0, 30, and 60 minutes of magnetization, under 40% compressive strain applied in the vertical direction at a velocity of 5 mm s^−1^.


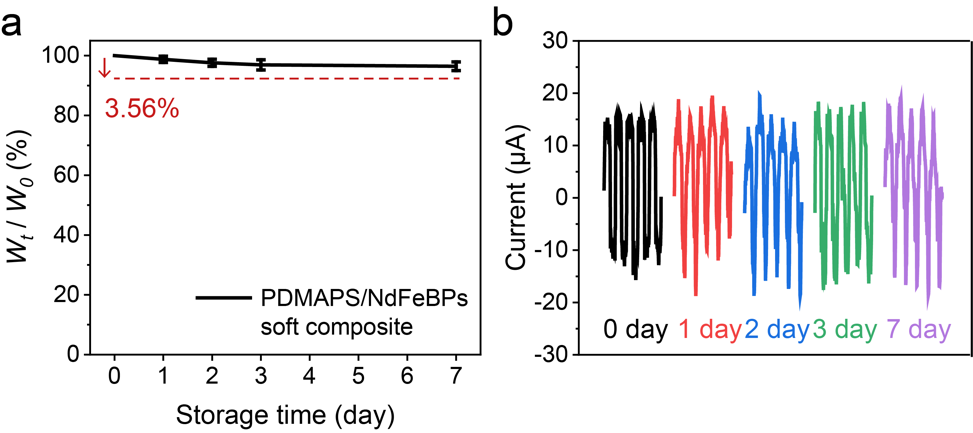


**Figure S23.** Long-term stability of the PDMAPS/NdFeBPs soft composite. a) Mass content retention over 7 days (*n* = 3; error bars represent the SD). b) Time-dependent stability of the PDMAPS/NdFeBPs composite under 40 % strain at 5 mm s^-1^.





**Figure S24.** All letters are indicated in Morse Code.


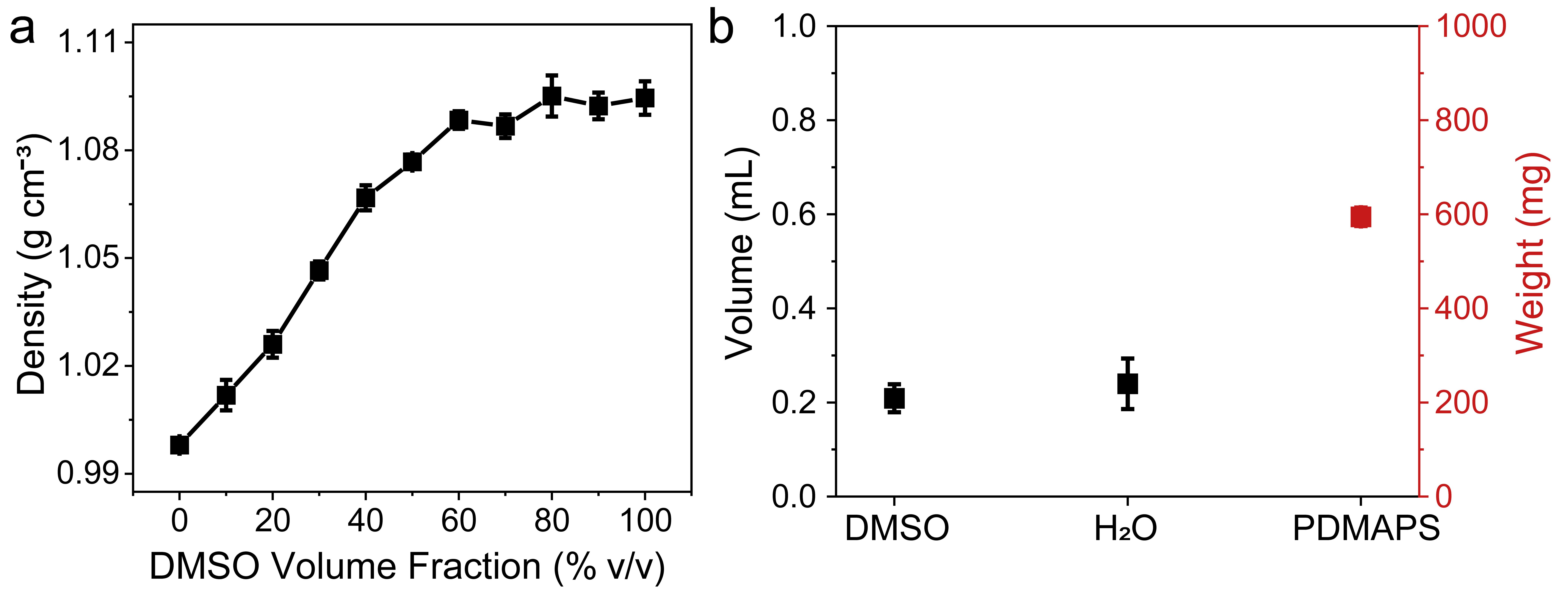


**Figure S25.** a) Density measured with varying DMSO volume fractions (%, v/v) in the DMSO–water mixture. b) The compositions of DMSO (volume), water (volume), and PDMAPS (weight mass) in a PDMAPS soft composite prepared with 29% (v/v) DMSO. Sample size *n* = 3; error bars correspond to SD. The notation v/v denotes the volume of DMSO as a fraction of the total volume of the prepared mixture.


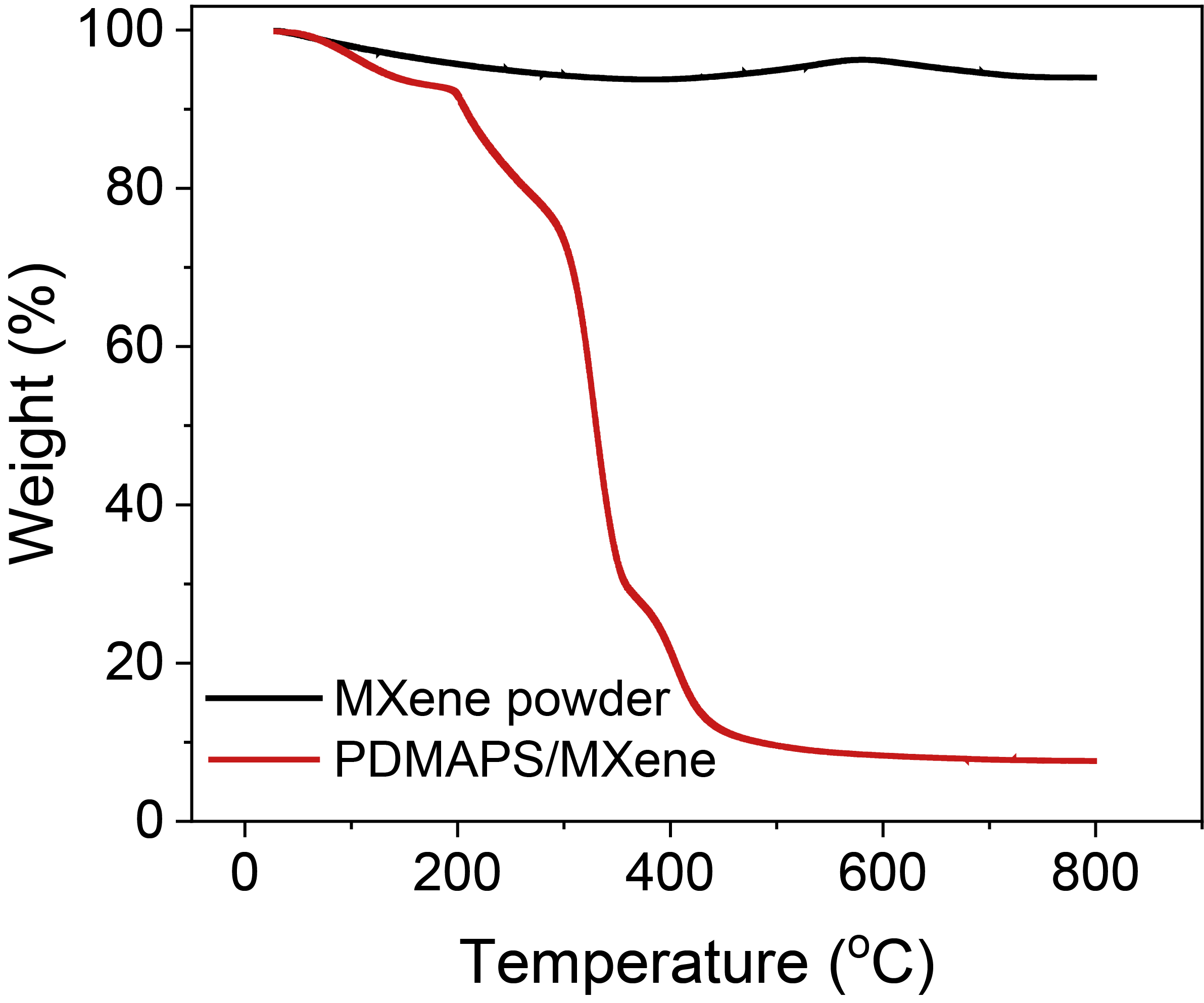


**Figure S26.** TGA curve of the lyophilized PDMAPS/MXene composite, showing a residual mass of 7.62 wt.% at 800 °C. After correcting for solvent removal, the estimated MXene content was approximately 5.94 wt.%.


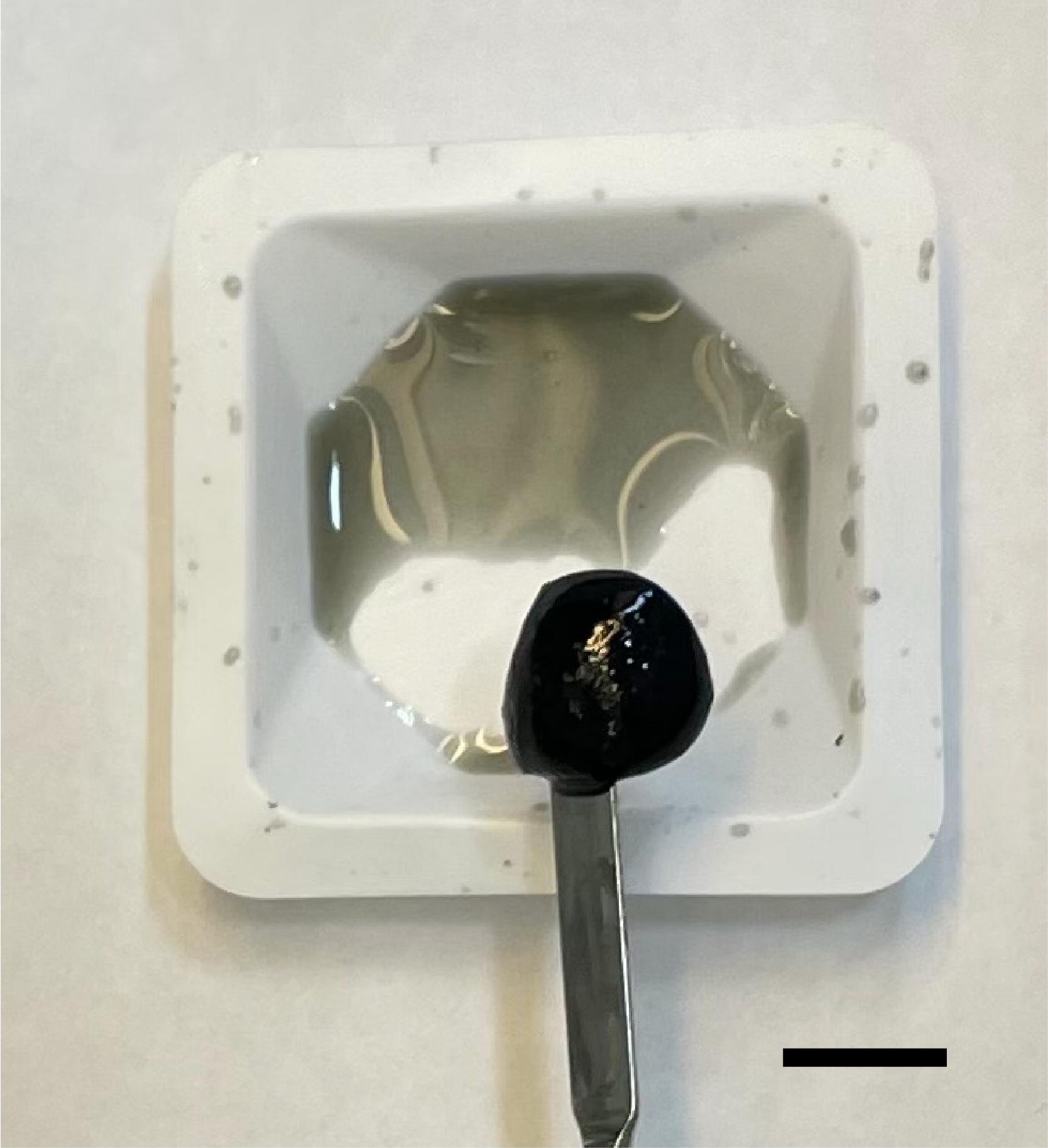


**Figure S27.** Photograph of the removed solvent and the corresponding PDMAPS/MXene composite after preparation, showing a nearly transparent solvent phase, which indicates negligible MXene residue and confirms complete incorporation of MXene into the PDMAPS matrix. (Scale bar: 1 cm)

**Movie S1.** Formation process of PDMAPS-based organohydrogels.

**Movie S2.** Dynamic demonstration of the self-adhesive/healing process.

**References**

1. Li P, Wang H, Ju Z, Jin Z, Ma J, Yang L, et al. “Ti_3_C_2_T_x_ MXene- and Sulfuric Acid-Treated Double-Network Hydrogel with Ultralow Conductive Filler Content for Stretchable Electromagnetic Interference Shielding,” *ACS Nano* 18, no. 4 (2024): 2906-2916. <https://doi.org/10.1021/acsnano.3c07233>

2. Yu Y, Yi P, Xu W, Sun X, Deng G, Liu X, et al. “Environmentally Tough and Stretchable MXene Organohydrogel with Exceptionally Enhanced Electromagnetic Interference Shielding Performances,” *Nano-micro Letters* 14, no. 1 (2022): 77. <https://doi.org/10.1007/s40820-022-00819-3>

3. Mei L, Ouyang W, Xu L, Huang Y, Liu Q, Bai Y, et al. “Super Tough Multifunctional MXene/PAA‐CS Double Network Hydrogels with High Mechanical Sensing Properties and Excellent EMI Shielding Performance,” *Small* 21, no. 6 (2025): 2410687. <https://doi.org/10.1002/smll.202410687>

4. Yang Y, Wu N, Li B, Liu W, Pan F, Zeng Z, et al. “Biomimetic Porous MXene Sediment-Based Hydrogel for High-Performance and Multifunctional Electromagnetic Interference Shielding,” *ACS Nano* 16, no. 9 (2022): 15042-15052. <https://doi.org/10.1021/acsnano.2c06164>

5. Yan Q, Liu Z, Xiong J, Lian H, Chen H, Fei T, et al. “Stimuli‐Responsive MXene/PNIPAM Hydrogel With High‐Performance and Tunable Electromagnetic Interference Shielding Performance,” *Advanced Science* (2025): e05551. <https://doi.org/10.1002/advs.202505551>

6. Yang Y, Li B, Wu N, Liu W, Zhao S, Zhang CJ, et al. “Biomimetic Porous MXene-Based Hydrogel for High-Performance and Multifunctional Electromagnetic Interference Shielding,” *ACS Materials Letters* 4, no. 11 (2022): 2352-2361. <https://doi.org/10.1021/acsmaterialslett.2c00778>
